# Reactor microbiome enriches vegetable oil with n-caproate and n-caprylate for potential functionalized feed additive production via extractive lactate-based chain elongation

**DOI:** 10.1186/s13068-021-02084-9

**Published:** 2021-12-06

**Authors:** Carlos A. Contreras-Dávila, Norwin Zuidema, Cees J. N. Buisman, David P. B. T. B. Strik

**Affiliations:** Environmental Technology, Wageningen University & Research, Bornse Weilanden 9, 6708 WG Wageningen, The Netherlands

**Keywords:** Reactor microbiomes, Microbiome engineering, *Caproiciproducens*, Extractive chain elongation, Medium-chain carboxylates, Lactate, Liquid–liquid extraction, Sunflower oil, Oleyl alcohol

## Abstract

**Background:**

Biotechnological processes for efficient resource recovery from residual materials rely on complex conversions carried out by reactor microbiomes. Chain elongation microbiomes produce valuable medium-chain carboxylates (MCC) that can be used as biobased starting materials in the chemical, agriculture and food industry. In this study, sunflower oil is used as an application-compatible solvent to accumulate microbially produced MCC during extractive lactate-based chain elongation. The MCC-enriched solvent is harvested as a potential novel product for direct application without further MCC purification, e.g., direct use for animal nutrition. Sunflower oil biocompatibility, in situ extraction performance and effects on chain elongation were evaluated in batch and continuous experiments. Microbial community composition and dynamics of continuous experiments were analyzed based on 16S rRNA gene sequencing data. Potential applications of MCC-enriched solvents along with future research directions are discussed.

**Results:**

Sunflower oil showed high MCC extraction specificity and similar biocompatibility to oleyl alcohol in batch extractive fermentation of lactate and food waste. Continuous chain elongation microbiomes produced the MCC n-caproate (nC6) and n-caprylate (nC8) from l-lactate and acetate at pH 5.0 standing high undissociated n-caproic acid concentrations (3 g L^−1^). Extractive chain elongation with sunflower oil relieved apparent toxicity of MCC and production rates and selectivities reached maximum values of 5.16 ± 0.41 g nC6 L^−1^ d^−1^ (MCC: 11.5 g COD L^−1^ d^−1^) and 84 ± 5% (e^−^ eq MCC per e^−^ eq products), respectively. MCC were selectively enriched in sunflower oil to concentrations up to 72 g nC6 L^−1^ and 3 g nC8 L^−1^, equivalent to 8.3 wt% in MCC-enriched sunflower oil. Fermentation at pH 7.0 produced propionate and n-butyrate instead of MCC. Sunflower oil showed stable linoleic and oleic acids composition during extractive chain elongation regardless of pH conditions. Reactor microbiomes showed reduced diversity at pH 5.0 with MCC production linked to *Caproiciproducens* co-occurring with *Clostridium* *tyrobutyricum*, *Clostridium* *luticellarii* and *Lactobacillus* species. Abundant taxa at pH 7.0 were *Anaerotignum*, *Lachnospiraceae* and *Sporoanaerobacter*.

**Conclusions:**

Sunflower oil is a suitable biobased solvent to selectively concentrate MCC. Extractive reactor microbiomes produced MCC with improved selectivity and production rate, while downstream processing complexity was reduced. Potential applications of MCC-enriched solvents may include feed, food and biofuels purposes.

**Supplementary Information:**

The online version contains supplementary material available at 10.1186/s13068-021-02084-9.

## Background

Many biotechnological processes for resource recovery from residual materials are being developed based on reactor microbiomes. Reactor microbiomes are capable of carrying out complex conversions as compared to pure cultures to produce valuable products, such as methane, carboxylic acids or polyhydroxyalkanoates from non-sterile residual substrates [1]. Selection pressures (e.g., temperature, pH, solids or liquid retention times) applied to adapted environments (reactors) are central for steering bioprocesses [2] and engineering reactor microbiomes [3]. Understanding the factors governing reactor microbiomes assembly and functioning will help harness the full potential of microbiomes [1, 3]. Novel resource recovery bioprocesses using chain elongation microbiomes produce medium-chain carboxylates (MCC), saturated monocarboxylic acids with 6 to 12 carbon atoms that find applications in lubricants, biodegradable plastics, antimicrobials, feed additives and biofuels production [2]. Chain-elongating bacteria utilize energy-rich substrates (e.g., ethanol, lactate, sugars) as electron donors to elongate short-chain carboxylates (SCC, 1–5 carbon units) to MCC such as n-caproate (nC6) or n-caprylate (nC8) through a series of biochemical reactions in the reverse β-oxidation pathway [2]. These electron donors can be obtained from low-cost organic residual materials to achieve MCC production directly from the residues [4, 5] or with externally added electron donors [6, 7]. When lactate is the electron donor used, mildly acidic conditions are typically needed to achieve chain elongation to MCC [8, 9]. The produced MCC should then be separated from the fermentation broth for their valorization. At lab-scale, methods such as liquid–liquid extraction [8, 10] or ion exchange [11] have been developed for MCC separation and have resulted in improved chain elongation, including lactate conversion to n-caproate [8]. Other alternative methods for carboxylates (referred here as the sum of their undissociated and dissociated forms) separation, include precipitation, adsorption or extractive fermentation [12]. Extractive fermentation is the process, where the broth (aqueous phase) is contacted with a solvent (organic phase) during fermentation, resulting in simultaneous production and in situ recovery of fermentation products. Adequate extractive fermentation may decrease product inhibition, chemicals input for pH control and increase bioprocess effectivity [12]. Solvent biocompatibility is one first prerequisite to attain successful extractive fermentation, with biocompatibility meaning that the solvent must not hamper the pertinent bioprocess [13]. Other important criteria are: high selectivity towards the product, low solubility in broth, high carboxylates recovery at low concentrations, low-cost and suitable physical properties for phase separation [12, 14]. Mineral oil is a fossil-based solvent frequently used in chain elongation, although not known to be biocompatible, since it is used through membrane-based separation processes [8, 10]. On the other hand, oleyl alcohol is a commonly used solvent shown to be biocompatible with different microbiomes [14, 15], including chain-elongating bacteria [16, 17]. Alternatively, vegetable oils containing triglycerides of long-chain carboxylates (LCC) may be used as biobased solvents [18]. In a previous study, MCC seemed to be partly extracted by the oil contained in food waste during chain elongation [4]. Although vegetable oils have been tested for ethanol extraction [18], their MCC extraction performance and biocompatibility with chain-elongating microbiomes have not been reported. In this work, the terms extractive fermentation and extractive chain elongation are used interchangeably.

Vegetable oils are envisioned here as promising matrixes to accumulate MCC during extractive chain elongation for the direct application of MCC-enriched oils after being harvested or skimmed off the reactor. Extractive fermentation with application-compatible solvents can lead to novel products from microbial chain elongation while impacting the process positively by, for instance, increasing fermentation efficiency and reducing downstream processing (DSP) complexity. Typically, further steps are needed for solvent regeneration and MCC purification after MCC are concentrated [19]. Solvent regeneration is usually done through back-extraction with strong inorganic bases which requires additional chemicals and generates waste inorganic salts [12]. Purification may be done by energy-demanding processes, such as distillation [20] or membrane electrolysis [19], adding to DSP complexity. At industrial scale, even a six-steps down stream processing (DSP) based on physical separation and evaporation techniques was proposed by ChainCraft B.V. to recover MCC salts obtained from food waste and ethanol for animal nutrition applications [21]. Alternatively, application-compatible solvents to avoid product-solvent separation has been deemed attractive for advanced biofuels fermentation processes [22] with benefits including reduced production costs and environmental footprint [23]. One potential application of MCC-enriched vegetable oils is their use as novel food or feed additives with diverse functionalities. MCC display differential effects on human health compared to unsaturated LCC [24]. Vegetable oils and the LCC contained in them are shown to have positive effects in livestock growth [25, 26], while MCC can be used in low doses to inactivate pathogens in feed and improve swine health and performance [27]. Both LCC and MCC can be used as natural alternatives to antibiotics [26, 27] and for methane mitigation in cattle [28–30].

Thus, this work aims to assess the feasibility of producing MCC-enriched vegetable oil via lactate-based chain elongation. Extraction capability and biocompatibility of sunflower oil, a widely available vegetable oil, is compared against oleyl alcohol in batch extractive fermentation using lactate and food waste as substrates. Then, continuous bioreactor experiments were performed to evaluate extractive chain elongation of l-lactate and acetate and MCC accumulation in sunflower oil. Changes in microbiome composition and taxa differential abundance were studied using 16S rRNA gene sequencing data. MCC extraction with sunflower oil was compared using a synthetic effluent in abiotic continuous reactors.

## Results

### Sunflower oil and oleyl alcohol biocompatibility and carboxylates extraction efficiency in batch extractive chain elongation

**Fig. 1:**
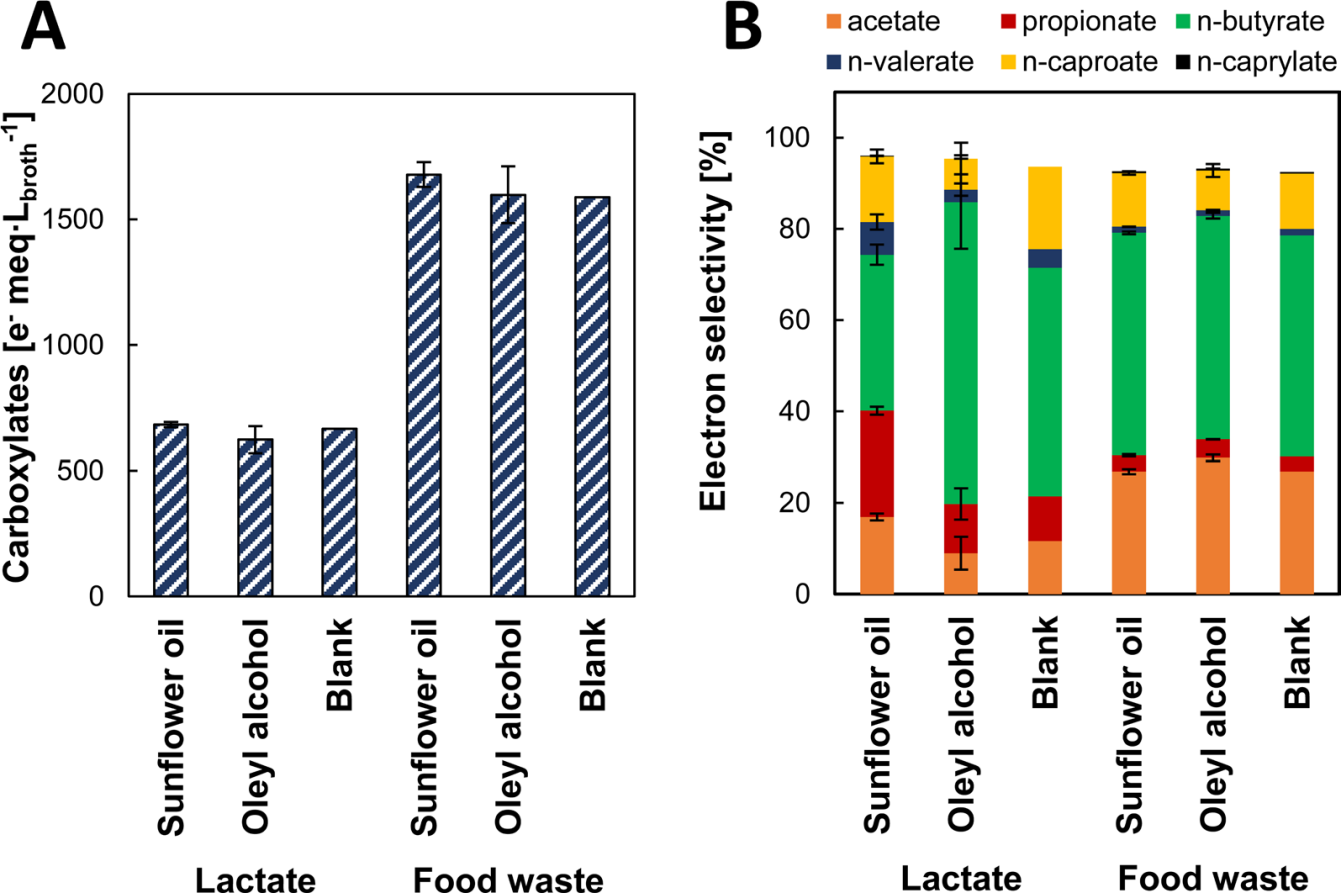
**A** Total carboxylates produced and **B** product selectivity from lactate and food waste (non-)extractive 20-day batch fermentation. Carboxylates partitioned in the organic and aqueous phases were added up and normalized to the initial aqueous phase (fermentation broth) volume. Error bars depict duplicates absolute deviation from the average

First, batch experiments were carried out to compare the biocompatibility and extraction performance of sunflower oil and oleyl alcohol in batch extractive fermentation using an inoculum from food waste fermentation [4]. After 20 days of incubation, experiments with sunflower oil and oleyl alcohol resulted in similar amounts of carboxylates produced compared to the blank experiments without solvent (Fig. 1A). A short delay in the exponential phase was observed for both solvents in the lactate experiments although this was not the case with food waste as substrate (Additional file 1: Fig. S1). These observations show the biocompatibility of both solvents with chain-elongating microbiomes, since the process occurred at similar carboxylates yield despite direct contact with the solvents. However, solvent choice affected product distribution (Fig. 1B). While n-butyrate (nC4) was the main fermentation product, extraction with sunflower oil favored the formation of n-caproate (nC6) from both substrates (lactate: 98 ± 12 e^−^ meq L^−1^; food waste: 196 ± 11 e^−^ meq L^−1^) when compared to oleyl alcohol (lactate: 44 ± 25 e^−^ meq L^−1^; food waste: 140 ± 33 e^−^ meq L^−1^) (Additional file 1: Table S1). Although n-caproate selectivities were similar (sunflower oil with lactate [14 ± 2%] and food waste [12 ± 1%] vs oleyl alcohol with lactate [7 ± 4%] and food waste [9 ± 1%]). In addition, sunflower oil addition to lactate-fed experiments showed higher acetate, propionate and n-valerate (nC5) formation with n-valerate reaching a selectivity of 7 ± 2% (Fig. 1B). n-caprylate (nC8) production occurred in low amounts from food waste (< 0.4% selectivity) showing no improvement with extraction. Sunflower oil extracted n-caproate preferably over n-butyrate, whereas the opposite trend was observed for oleyl alcohol (Additional file 1: Fig. S2). Extraction efficiency under fermentation conditions is compared for both solvents in Table 1. Oleyl alcohol displayed overall higher *K*_D_ (1–6 times) and recovery (1.5–3.8 times) for both n-butyrate and n-caproate compared to sunflower oil. On the other hand, sunflower oil specificity for n-caproate was similar or higher than that of oleyl alcohol. Extraction specificity for n-caproate was 1.8–2.4 higher than for n-butyrate with sunflower oil vs 0.7–1.0 for oleyl alcohol. The apparent low *K*_D_ for nC8 may be related to its production at trace concentrations from food waste (6–7 e^−^ meq L^−1^) (Additional file 1: Table S1).

**Table 1:** Average sunflower oil and oleyl alcohol extraction efficiency during extractive fermentation

Parameter	Carboxylate	Lactate		Food waste	
		Sunflower oil	Oleyl alcohol	Sunflower oil	Oleyl alcohol
Final pH		6 ± 0.1	6.5 ± 0.1	4.8 ± 0	4.9 ± 0
*K*_D_ (distribution ratio)	nC4	0.1 ± 0	0.1 ± 0	0.2 ± 0	1 ± 0
	nC5	0.2 ± 0	0.2 ± 0.2	0.6 ± 0	2.9 ± 0.1
	nC6	0.5 ± 0.1	1.5 ± 0.3	2.5 ± 0	14.9 ± 0.4
	nC8	n.a	n.a	1.1 ± 0.1	1.1 ± 0.1
*P* (partition coefficient)	nC4	1 ± 0.3	0.2 ± 0	2.6 ± 0.1	0.4 ± 0
	nC5	0.4 ± 0.1	0.1 ± 0	0.9 ± 0	0.1 ± 0
	nC6	0.1 ± 0	0 ± 0	0.2 ± 0	0 ± 0
	nC8	n.a	n.a	0.5 ± 0	0.5 ± 0.1
Recovery [%]	nC4	2.3 ± 0.3	2.7 ± 0.7	5.6 ± 0.1	23.4 ± 0.3
	nC5	5.7 ± 0.8	5.9 ± 5.9	15.4 ± 0.3	48.1 ± 1.1
	nC6	14.4 ± 1.4	31.4 ± 5.2	44.5 ± 0.1	82.5 ± 0.4
	nC8	n.a	n.a	26.3 ± 1.8	25 ± 2.1
Specificity [%]	nC4	18.2 ± 1	43.6 ± 13.8	30.3 ± 0.1	50.2 ± 3
	nC5	8.6 ± 1.9	3.4 ± 3.4	2.1 ± 0	2.6 ± 0.1
	nC6	44.1 ± 14.5	41.4 ± 12.2	54.9 ± 0	32.8 ± 3.7
	nC8	n.a	n.a	1 ± 0.1	0.3 ± 0

### Continuous lactate-based chain elongation with(out) sunflower oil

**Fig. 2:**
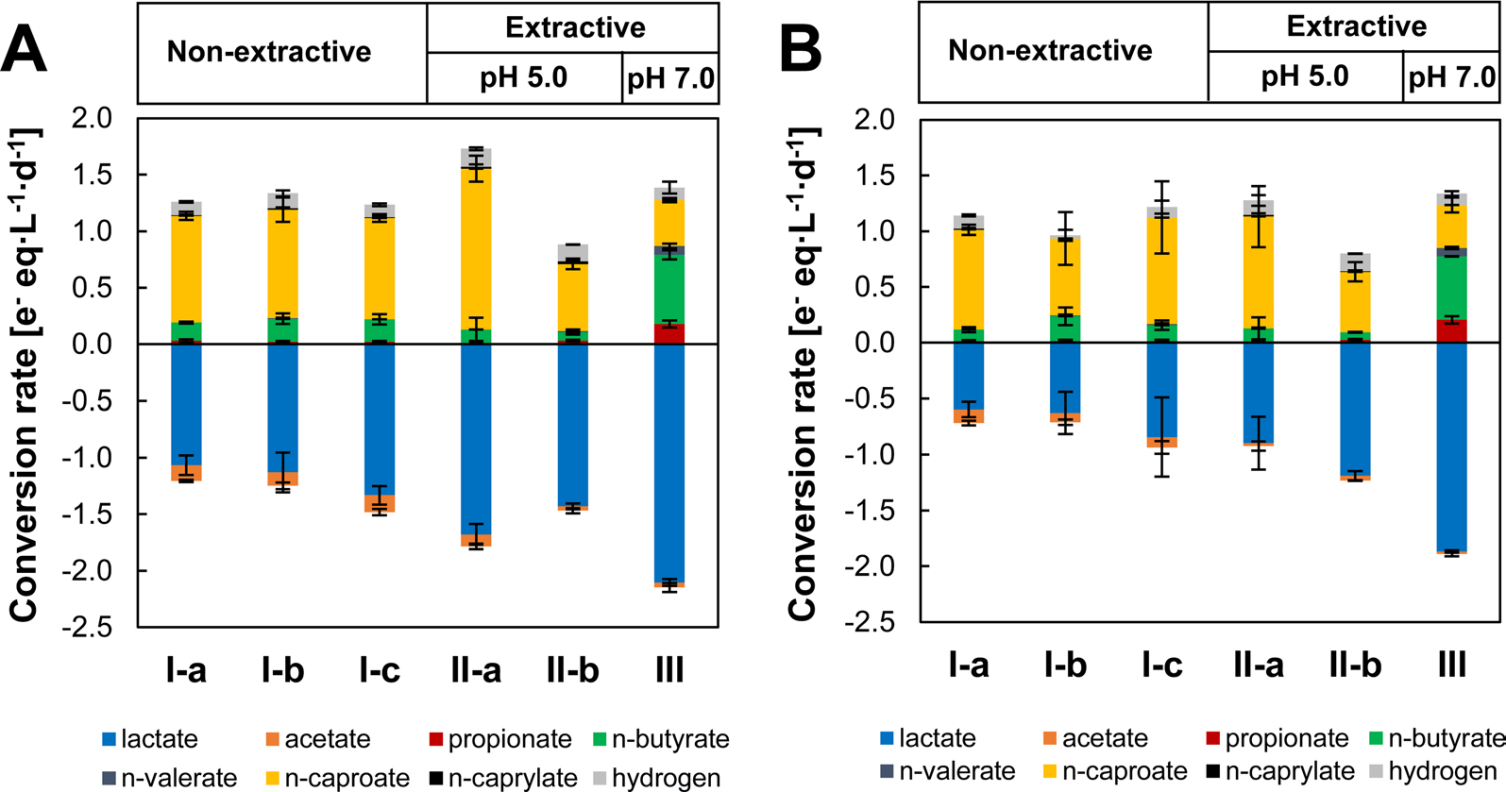
Conversion rates in (non-)extractive chain elongation continuous reactors: **A** R1 and **B** R2. Initial substrates were l-lactate and acetate. Conversion rates include carboxylates in aqueous and organic phases normalized to reactors working volume. Error bars show ± one standard deviation except for periods II-a and III for which absolute deviation is shown

To prove the capability to enrich sunflower oil with MCC, continuous chain elongation experiments were carried out in two independent 2-L CSTR fed with l-lactate and acetate using the same inoculum as in batch extractive fermentation (“Sunflower oil and oleyl alcohol biocompatibility and carboxylates extraction efficiency in batch extractive chain elongation”). Fermentation performance was first evaluated in the absence of sunflower oil at constant pH 5.0 and 2 d HRT. Under these conditions, n-caproate was the dominant product with both reactors showing similar performance throughout the operation. The overview of both reactors performance can be found in Additional file 1: Fig. S3, Tables S2 and S3. After ~ 20 days of adaptation, n-caproate was steadily produced at rates of 943.5 ± 36 e^−^ meq L^−1^ d^−1^ (3.43 ± 0.13 g L^−1^ d^−1^) in R1 and 894 ± 46 e^−^ meq L^−1^ d^−1^ (3.24 ± 0.17 g L^−1^ d^−1^) in R2 during the period I-a (Fig. 2). Production of MCC (nC6-nC8) was 969 ± 63 e^−^ meq L^−1^ d^−1^ and 921 ± 36 e^−^ meq L^−1^ d^−1^ in R1 and R2, respectively. n-Caproate was the main product of chain elongation with electron selectivities of 74 ± 2% (76 ± 1% MCC) in R1 and 77 ± 2% (80 ± 2% MCC) in R2. n-Butyrate and hydrogen were the main side-products (~ 10% electron selectivity each) and selectivity for the MCC n-heptanoate and n-caprylate was ~ 1% for each (Additional file 1: Tables S2, S3). n-Caproate concentrations were ~ 6.5 g L^−1^ and about half of the lactate fed (47–64%) remained unconsumed. After period I-a, technical complications caused pH to temporarily reach near-neutral values (pH 7.0–7.4) with higher lactate consumption and n-caproate concentrations (8.2–14.5 g L^−1^) observed (Additional file 1: Fig. S4). Manual acidification of the medium back to pH 5.0 resulted in biomass wash out (R1 day 45.8; R2 day 46.8). The reactors were operated in batch mode with no pH control to allow biomass regrowth. Once biomass growth was observed and pH stabilized at ~ 7.3 (~ 5 days), continuous operation was reinitiated and pH was readjusted in a stepwise manner by automatic acid addition (0.5 units every 0.5–1 HRT). The acid input in period I-a was 67–76 mmol H^+^ L^−1^ d^−1^ and 2.4–2.5 mol H^+^ mol MCC^−1^ (Additional file 1: Tables S2, S3). Both CSTR recovered n-caproate production after these perturbations. However, R2 showed high variability in n-caproate production rates thereafter (Additional file 1: Fig. S3B) expressed by unstable oxidation–reduction potential (ORP). ORP increased from − 405 ± 8 mV in period I-a to − 367 ± 19 mV in period I-b and varied around − 400 ± 45 mV in period I-c (Additional file 1: Table S3). In contrast, ORP remained constantly lower in R1, between − 475 ± 8 and − 496 ± 1 mV during non-extractive chain elongation (Additional file 1: Table S2). Methane was only sporadically produced (< 5 mmol L^−1^ d^−1^) during R2 batch recovery from biomass wash out.

**Fig. 3:**
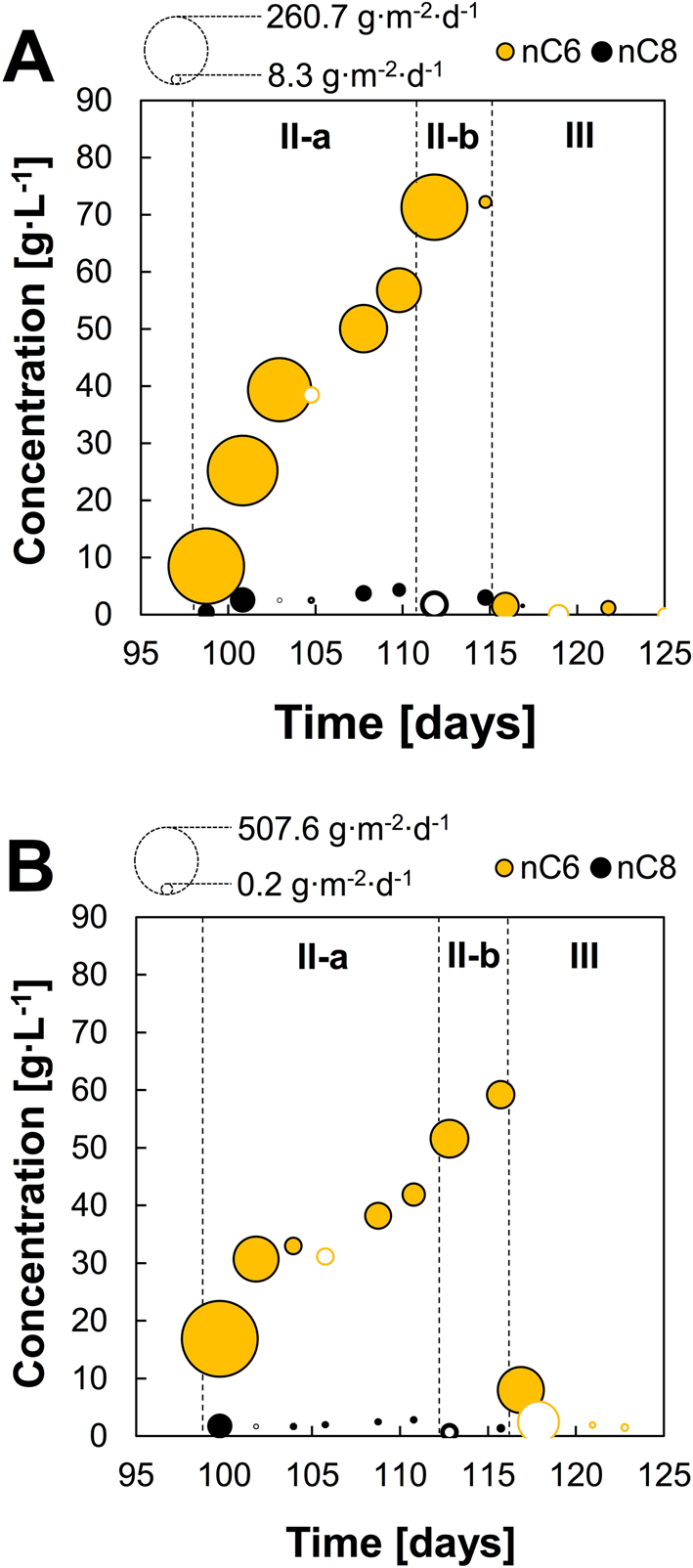
Medium-chain carboxylates concentration in sunflower oil as measured in back-extracted samples from extractive chain elongation continuous reactors: **A** R1 and **B** R2. Initial substrates were l-lactate and acetate. Bubble size shows extraction flux to the solvent based on cumulative carboxylates concentrations between two contiguous sampling points. Empty bubbles show negative flux values (apparent re-solubilization to the aqueous phase). Operational days corresponding to periods II-a, II-b and III are shown in Additional file 1: Table S1

In extractive chain elongation, sunflower oil was present on top of the fermentation broth. The solvent phase was above the stirring propeller level resulting in only slight mixing due to stirring turbulence and biogas bubbling up to the headspace. During extractive fermentation, aqueous phase HRT and stirring speed operational conditions were kept constant. Lactate conversion rates increased after addition of sunflower oil with no apparent adaptation phase. n-Caproate production rates were enhanced with sunflower oil addition in both reactors but this effect was maintained for over 5 HRTs only in R1 (period II-a) at 1421 ± 114 e^−^ meq L^−1^ d^−1^ (5.16 ± 0.41 g L^−1^ d^−1^) (Additional file 1: Table S2). After this period, a drop in n-caproate production (542 ± 86 e^−^ meq L^−1^ d^−1^; 1.97 ± 0.32 g L^−1^ d^−1^) and extraction rates was observed (period II-b). R2 performance remained unstable during period II-a with no clear improvement in n-caproate production rates and a similar decrease in n-caproate production was observed in period II-b (Fig. 2). n-Caproate and n-caprylate accumulated in the sunflower oil to concentrations of 72 and 3 g L^−1^, respectively, by the end of period II-a in R1. These concentrations were 59 g L^−1^ for n-caproate and 1.3 g L^−1^ for n-caprylate in R2 (Fig. 3). The highest measured extraction rates were 1.9 g nC6 L_broth_^−1^ d^−1^ in R1 (260.7 g nC6 m^−2^ d^−1^) and 3.7 g nC6 L_broth_^−1^ d^−1^ (507.6 g nC6 m^−2^ d^−1^) in R2 (Fig. 3). Under these conditions, n-caproate and n-caprylate were selectively extracted with no other SCC or MCC detected in sunflower oil. This was confirmed in abiotic continuous experiments, where n-caproate from synthetic effluent was the only carboxylate extracted into sunflower oil, while lactate, acetate and n-butyrate remained in the effluent (Additional file 1: Fig. S5). Maximum extraction rates in the abiotic experiment were around half those observed during extractive chain elongation (1.1–1.7 g nC6 L_broth_^−1^ d^−1^; 149–236 g nC6 m^−2^ d^−1^), probably due to the higher chain elongation rates and consequent n-caproate loadings during extractive fermentation (Fig. 2).

Extractive chain elongation also improved n-caproate electron selectivity in R1 from 71 ± 3% during non-extractive chain elongation (average periods I-a to I-c) to 83 ± 6% (period II-a). Likewise, n-caproate carbon selectivity increased from 50 ± 3% to 58 ± 3%. Carbon conversion efficiency towards n-caproate improved from period I-c (35 ± 2%) to period II-a (45 ± 3%) but was not different from the average of non-extractive chain elongation periods (I-a to I-c; 40 ± 4%) (Additional file 1: Table S2). For the case of R2, improvement in the aforementioned parameters was not clear due to reactor instability e g. n-caproate electron selectivity was 76 ± 6% during non-extractive chain elongation (periods I-a to I-c) compared to 80 ± 8% during period II-a (Additional file 1: Table S3). Recovery in sunflower oil was limited to 15–18% of the n-caproate flux, while the totality of n-caprylate partitioned to the organic phase (Additional file 1: Fig. S6).

Before period III, sunflower oil was collected from the reactors, fermentation broth pH adjusted to 7.0 and new sunflower oil added. The highest substrate conversion rates were observed under this neutral pH conditions for both reactors (Fig. 2). Nevertheless, n-caproate production declined over time and increasing n-butyrate, propionate and n-valerate were observed (Additional file 1: Fig. S3). Electron selectivity in R1 decreased to 28 ± 3% for n-caproate, while it reached 44 ± 2% for n-butyrate, 13 ± 2% for propionate and 6 ± 2% for n-valerate. Similar metabolites profile and selectivities were observed in R2. n-Caproate was still extracted into sunflower oil at pH 7.0 but it was re-solubilized into the aqueous phase as n-caproate production declined over time (Fig. 3, Additional file 1: Fig. S6).

### MCC-enriched sunflower oil composition

About 80% of the added sunflower oil was recovered from the reactors after extractive fermentation. The rest was lost during the manual harvesting procedure (“Continuous lactate-based chain elongation and extraction with sunflower oil**”**). The respective carboxylic acids composition before and after extractive fermentation are shown in Table 2. From the back-extracted samples measured in our lab, MCC were estimated to make up 6–8.6% of the total carboxylic acids in MCC-enriched sunflower oil with nC8-to-nC6 carbon ratios of 2–4% (this ratio was ~ 1% in the effluent). Principal components analysis (PCA) of C4–C23 carboxylic acids proportions (ISO 15885) [31] showed that the overall sunflower oil composition between the original and the harvested sunflower oils was relatively similar (Additional file 1: Fig. S7). The proportions of unsaturated carboxylic acids (UCA) decreased due to accumulation of saturated carboxylic acids (SCA), mainly n-caproic acid (C6:0). Monounsaturated carboxylic acids (MUCA) proportions showed minor declines at pH 5.0 related with decreases in oleic acid content. Linoleic and oleic acids remained at similar proportions in sunflower oil after extractive fermentation. The actual proportions of UCA in general may be slightly lower in the MCC-enriched oil, since the ISO 15885 standard has been reported to underestimate SCC (nC4) and MCC (nC6 and nC8) [32]. This may explain the much lower MCC content obtained with the standard analysis compared to the back-extraction results from our lab. n-caproic and n-caprylic acids presence as free carboxylic acids instead of esterified forms may have also influenced their correct quantification.

**Table 2:** Carboxylic acids composition of sunflower oil before and after extractive chain elongation at different pH conditions

Compound	Initial sunflower oil	R1		R2	
		pH 5.0 (period II-b)	pH 7.0 (period III)	pH 5.0 (period II-b)	pH 7.0 (period III)
*Back-extraction* *estimations*					
% n-caproic acid (C6:0)^a^	0	8.20 ± 0.05	0.07 ± 0.07	6.33 ± 0.43	0.08 ± 0.08
% n-caprylic acid (C8:0)^a^	0	0.27 ± 0.07	0	0.11 ± 0.04	0
wt% MCC		8.22 ± 0.12	0.06 ± 0.06	6.48 ± 0.23	0.08 ± 0.08
nC8-to-nC6 [% mol C]	N.A	3.5 ± 0.1	N.A	1.9 ± 0.5	N.A
*ISO* *15885* *standard*					
% n-caproic acid (C6:0)	0	0.84 ± 0.11	0	0.40 ± 0.03	0
% n-caprylic acid (C8:0)	0	0	0	0	0
% Oleic acid (C18:1, cis 9)	34.85 ± 0.03	34.29 ± 0.1	35.08 ± 0.01	34.58 ± 0.21	35.07 ± 0.11
% Linoleic acid (C18:2, cis 9, 12)	52.92 ± 0.06	52.78 ± 0.22	53.14 ± 0.05	52.95 ± 0.18	53.06 ± 0.08
% Saturated carboxylic acids (SCA)	10.64 ± 0.02	11.34 ± 0.11	10.19 ± 0.02	10.95 ± 0.01	10.28 ± 0.21
% Unsaturated carboxylic acids (UCA)	89.36 ± 0.02	88.66 ± 0.11	89.81 ± 0.02	89.05 ± 0.01	89.72 ± 0.21
% Conjugated linoleic acids (CLA)	0	0	0	0	0
% Ω-3 carboxylic acids	0.14 ± 0.01	0.13 ± 0.01	0.14 ± 0.01	0.13 ± 0.01	0.13 ± 0.01
% Ω-6 carboxylic acids	52.92 ± 0.07	52.78 ± 0.22	53.14 ± 0.05	52.95 ± 0.18	53.06 ± 0.08
% Monounsaturated carboxylic acids (MUCA)	35.74 ± 0.02	35.16 ± 0.10	35.95 ± 0.02	35.43 ± 0.20	35.95 ± 0.12
% Polyunsaturated carboxylic acids (PUCA)	53.06 ± 0.6	52.91 ± 0.21	53.28 ± 0.04	53.08 ± 0.19	53.19 ± 0.08
% Unnamed carboxylic acids	0.56 ± 0.03	0.59 ± 0.01	0.58 ± 0.01	0.54 ± 0.01	0.58 ± 0.01

Values from last two samples of each operational period are averaged

Error represents average absolute deviation from actual values (*n* = 2)

^a^Assuming carboxylic acids make 97 wt% of sunflower oil

### *Caproiciproducens* species dominate MCC-producing chain elongation microbiomes

**Fig. 4:**
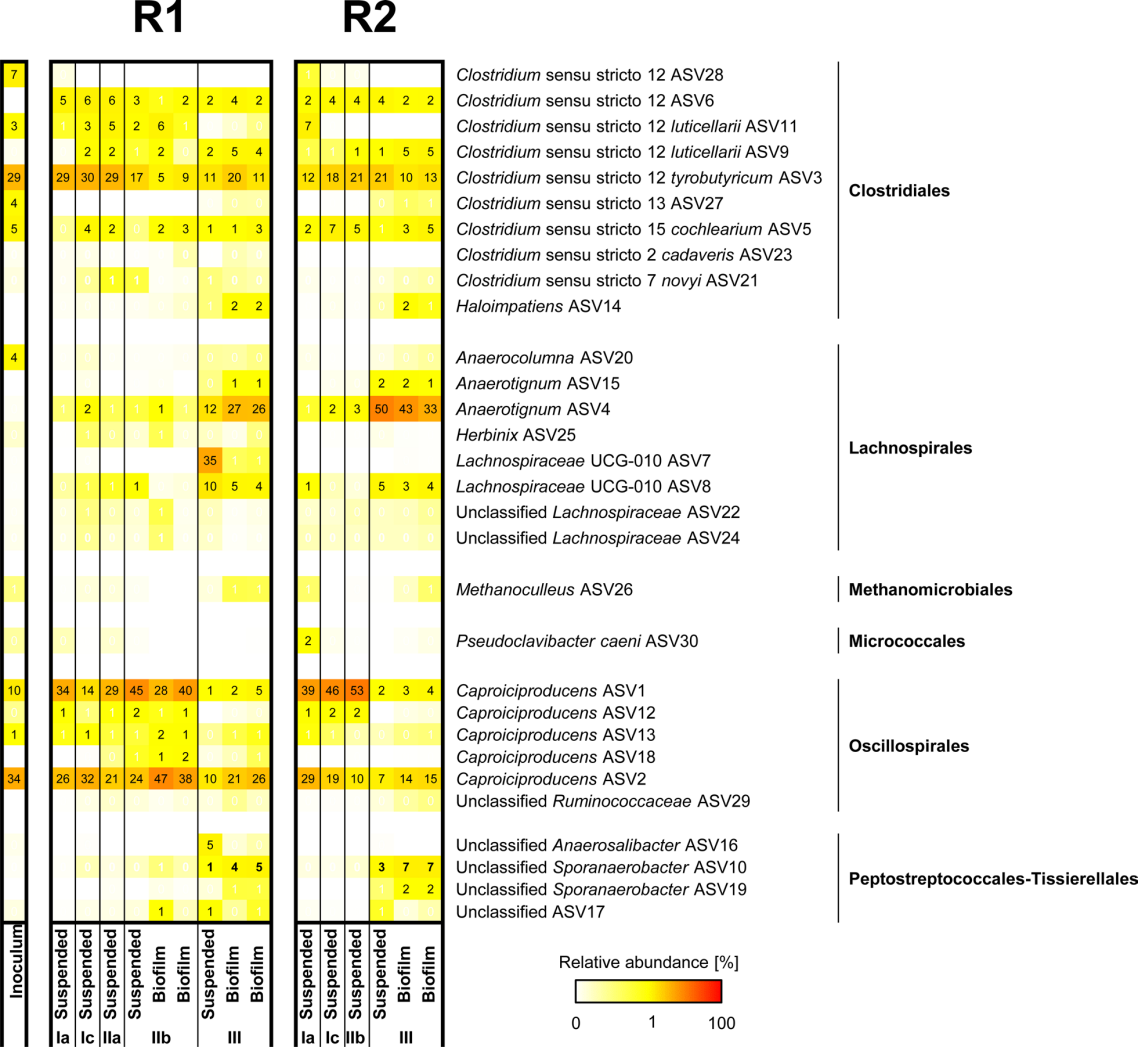
Microbial community composition in (non-)extractive chain elongation continuous reactors at ASV level. Initial substrates were l-lactate and acetate. Top 30 ASVs are displayed and relative abundance values ≥ 1% are shown. Independent biofilm samples were analysed and shown as duplicates. Sampling days are shown in Additional file 1: Fig. S4. Taxonomy was assigned based on SILVA 138 SSU Ref NR 99 database

**Fig. 5:**
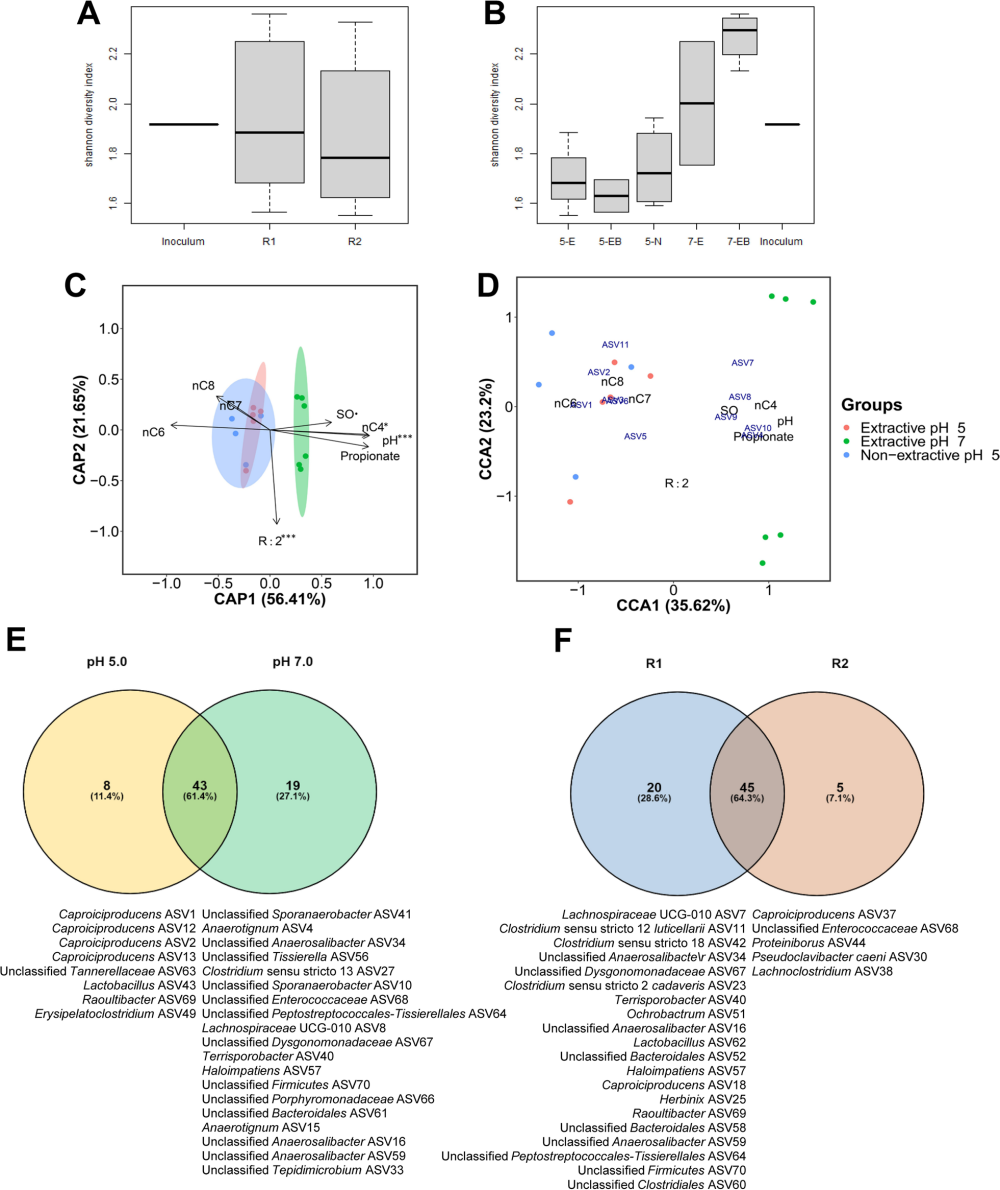
Microbial community dynamics in (non-)extractive chain elongation continuous reactors. Initial substrates were l-lactate and acetate. **A** Shannon diversity index of inoculum, R1 and R2 microbiomes. **B** Shannon diversity index of suspended microbiomes from non-extractive (5-N) and extractive chain elongation at pH 5.0 (5-E) and pH 7.0 (7-E); and biofilm microbiomes from extractive chain elongation at pH 5.0 (5-EB) and pH 7.0 (7-EB). Boxplots show the interquartile range (IQR) in boxes divided by median values (horizontal lines) with whiskers depicting ± 1.5 IQR (**A**, **B**). **C** Distance-based Redundancy Analysis (dbRDA) using Bray–Curtis dissimilarity index; ASVs relative abundance as response variables; and environmental parameters as explanatory variables (constraints). Environmental parameters considered were: reactor (R1, R2), pH, sunflower oil (SO) (presence/absence) and steady-state electron selectivities (propionate, nC4, nC6, nC7, nC8, MCC and H2; Additional file 1: Tables S2 and S3). Concentration ellipses depict confidence intervals with α = 0.05. Significance code: ‘***’ associated with a variable at *P* < 0.0005; ‘*’ associated with a variable at *P* < 0.01; and ‘.’ associated with a variable at *P* < 0.05. **D** Canonical Correspondence Analysis (CCA) using Hellinger transformation and same constraints as in dbRDA. CCA triplot shows top 11 ASVs scaled proportionally to eigenvalues. Collinear (redundant) environmental parameters (MCC and H_2_) were automatically dropped out in both dbRDA and CCA (**C**, **D**). **E**, **F** Differential abundance analyses at ASV level of microbiomes developed at different pH conditions (**E**) and between reactors (**F**). Differential abundance at different pH was analysed using all samples from both reactors. II-a and II-b biofilms samples were left out when comparing microbiomes between reactors. **E**, **F**. Analyses were done using CSS-normalized 16S rRNA gene sequencing data of ASVs with > 0.01% of total counts

Reactor microbiomes composition analysis based on 70 ASVs accounting for > 98.8% of counts showed that bacteria species from the orders *Clostridiales*, *Lachnospirales* and *Oscillospirales* accounted for 88–99% of reactors microbiomes with shifted proportions as pH was increased (Fig. 4). Both reactors showed similar Shannon diversity indexes throughout operation with microbiomes developed at pH 7.0 showing higher diversity compared to those developed at pH 5.0 (Fig. 5A, B). Biofilm microbiomes resembled suspended microbiomes composition in most of the cases although biofilms were more diverse at pH 7.0 compared to the corresponding suspended microbiomes. Reactor microbiomes composition was significantly affected by pH conditions (*P* < 0.0005) with weak influence of sunflower oil addition (*P* < 0.05) (Fig. 5C). *Caproiciproducens*-related ASVs were associated with MCC production and low pH conditions (Fig. 5D) and were differentially abundant at pH 5.0 (Fig. 5E). At this pH, *Caproiciproducens* was the most abundant genus under both non-extractive and extractive chain elongation conditions (48–82% relative abundance) followed by *Clostridium* sensu stricto 12 (12–42% relative abundance). Only a few ASVs could be assigned taxonomy at species level (100% match with SILVA database records), the rest are referred to by their ASV number and their similarity to BLAST records is reported. *Caproiciproducens* spp. ASV1 and ASV2 showed low similarity with the type strain *Caproiciproducens* *galactitolivorans* (< 91%) with the closest relative being [Clostridium] leptum strain DSM 753 (~ 93% similarity). *Anaerotignum*, *Lachnospiraceae* UCG-010 and *Sporoanaerobacter* related species were related with near-neutral pH conditions and formation of propionate and n-butyrate (Fig. 5C–E). *Anaerotignum* spp. ASV4 and ASV15 were 98.5 and 98% similar to *Anaerotignum* *propionicum*, respectively. The closest relative to *Lachnospiraceae* UCG-010 ASVs 7 and 8 was *Anaerotignum* *aminivorans* (95% similarity for both). Less abundant ASV10 and ASV19 were both > 99.5% similar to *Sporoanaerobacter* *acetigenes* DSM 13106. *Clostridium* *tyrobutyricum* spp. ASV3 and ASV6 (99.4% identity) were abundant throughout reactors operation and were both associated with MCC-producing microbiomes, while *Clostridium* *luticellarii* spp. ASV9 and ASV11 were related with propionate/n-butyrate- and MCC-producing microbiomes, respectively (Figs. 4, 5D). Sequences of selected ASVs can be found in Additional file 1: Table S4.

**Fig. 6:**
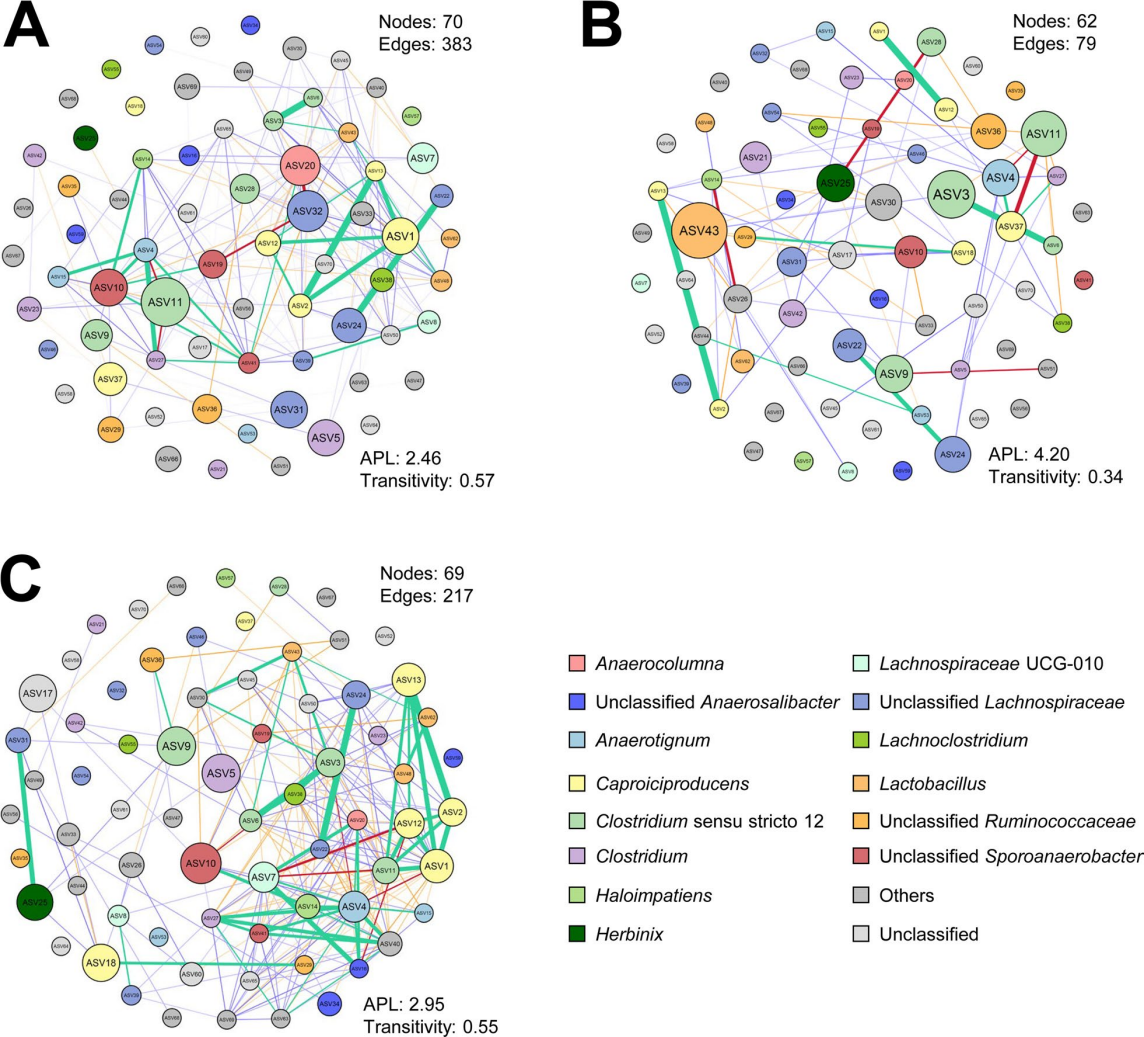
Co-occurrence networks of lactate-based chain elongation microbial communities. **A** Reactor microbiomes developed throughout reactors operation (*n* = 15); **B** at pH 5.0 (*n* = 9); and **C** throughout R1 operation (*n* = 9). Co-occurrence networks were built using significant correlations (*P* < 0.05). Strong correlations are depicted with green (> 0.8, positive correlation) and red (< − 0.8, negative correlation) edges with boldness related to the strength of the correlation. Positive and negative correlations with absolute values > 0.6 are shown with blue and orange edges, respectively, with increasing shading up to < 0.8. Nodes are sized based on ASVs betweenness centrality scores and coloured according to taxonomic classification at genus level

About 65% of the ASVs were equally enriched in both reactors with 20 ASVs in R1 and 5 ASVs in R2 being differentially abundant (Fig. 5F) which may help explain the more stable performance of R1. Of the differentially abundant taxa in R1, *Clostridium* *luticellarii* sp. ASV11 showed the highest betweenness centrality (BC) in the co-occurrence network constructed with samples from all operational periods (Fig. 6A) suggesting its relevance as hub microorganism in the microbiomes throughout reactors operation. In MCC-producing microbiomes developed at pH 5.0, *Lactobacillus* sp. ASV43, *Clostridium* *tyrobutyricum* sp. ASV3 and *Clostridium* *luticellarii* sp. ASV11 showed the highest BC values (Fig. 6B). Microbiomes from R1 showed highest BC for unclassified *Sporoanaerobacter* sp. ASV10, *C.* *luticellarii* sp. ASV9 and *C.* *cochlearum* sp. ASV5. R1 microbiomes show the co-occurrence of *Caproiciproducens* with *C.* *tyrobutyricum*, *C.* *luticellarii* sp. ASV11 and *Lactobacillus* (Fig. 6C).

## Discussion

### Lactate-based chain elongation microbiomes stand high undissociated n-caproic acid concentrations

Non-extractive continuous lactate-based chain elongation at pH 5.0 resulted in efficient production of MCC at an electron selectivity of 74 ± 4% (average R1 and R2). n-caproate was the main chain elongation product (98% of MCC) along with small but considerable amounts of n-heptylate and n-caprylate (~ 1% electron selectivity each). One study using continuously fed lactate reported n-caproate electron selectivities of 34% at pH 5.0 with n-heptylate but no n-caprylate formation [9]. Propionate production at high residual lactate concentrations has been reported to reduce chain elongation performance [8, 33]. We hypothesize that the accumulation of MCC at the acidic conditions applied in the present study prevented the dominance of propionate-producing bacteria in the reactor microbiomes, since robust chain elongation to n-caproate (~ 6.5 g L^−1^) and n-caprylate (~ 0.08 g L^−1^) proceeded at ~ 18 g L^−1^ residual lactate. Propionate formation was observed in the first days of operation. However, as n-butyrate was elongated, n-caproate concentrations increased gradually and propionate formation dropped when undissociated n-caproic acid concentrations reached values of ~ 1 g L^−1^ (8.8 mM). Stable n-caproate production and low propionate formation (~ 2% electron selectivity) were observed thereafter as these conditions led to reactor microbiomes with relatively low diversity specialized in MCC production. Uncultured *Caproiciproducens*-related microorganisms dominated MCC-producing microbiomes co-occurring with less abundant but highly connected (high betweenness centrality) hub microorganisms *Clostridium* *tyrobutyricum* sp. ASV3, *Clostridium* *cochlearium* sp. ASV5, *Lactobacillus* sp. ASV43 and *C.* *luticellarii* sp. ASV11. This suggests the relevance of low abundant organisms in the microbiomes structure and functioning, where niche complementarity may have supported the conversion of a larger fraction of substrate into MCC. In accordance to this study, *Caproiciproducens* species are reportedly identified to produce MCC from lactate [4, 9, 34]. *C.* *tyrobutyricum*, *C.* *cochlearium* and *Lactobacillus* may convert part of the lactate into n-butyrate [35] which may be further elongated by *Caproiciproducens*. *C.* *luticellarii* sp. ASV11 seems to be a strain adapted to mild acidic pH conditions supporting MCC production, while *C.* *luticellarii* sp. ASV9, identified to be strain FW431, was enriched in neutral pH conditions (Fig. 5D) and is reported to produce propionate from lactate [36]. Members of the *Ruminococcaceae* family such as *Caproiciproducens* seem to play an important role in the valorization of organic residues into MCC, since they have been enriched in other bioprocesses converting﻿ lactate [9, 34] or complex﻿ residues [4, 5].

Undissociated n-caproic acid is believed to be toxic to chain elongation microbiomes [37] at concentrations of 7.5 mM (0.87 g L^−1^) for ethanol-based processes [10]. However, *Caproiciproducens*-dominated microbiomes producing MCC from food waste with lactate as intermediary are reported to stand up to 20 mM undissociated n-caproic acid [4]. In the present study, increased lactate conversion to n-caproate was observed when pH increased momentarily in non-extractive chain elongation and when MCC were removed during extractive chain elongation. These observations suggest that undissociated n-caproic acid concentrations ~ 26 mM (3 g L^−1^) were limiting further lactate conversion to MCC and might represent threshold concentrations in efficient lactate-fed chain elongation systems. Moreover, complete inhibition of the microbiomes was observed when undissociated n-caproic acid concentrations reached 37 mM (4.3 g L^−1^) during manual pH readjustment (R1, day 46.8; R2, day 47.8). These threshold and inhibitory concentrations are 2 to 4 times higher than inhibitory concentrations proposed for ethanol-based chain elongation microbiomes [10]. However, further adaptation may make reactor microbiomes more resilient to MCC toxicity as shown for the ethanol-based chain elongation process [37].

### Extractive chain elongation improves bioprocess efficiency and rates

Removing part of the produced MCC via extractive chain elongation allowed the microbiomes to convert a larger fraction of the fed lactate resulting in 50–60% higher n-caproate production rates. The highest n-caproate volumetric production rate achieved here (R1 period II-a; 5.16 ± 0.41 g L^−1^ d^−1^) is ~ 65% higher than those obtained in a reactor fed l-lactate and n-butyrate equipped with in-line extraction [8]. Furthermore, extractive chain elongation increased n-caproate electron selectivity up to 83 ± 6% (84 ± 5% MCC; equivalent to 92% MCC per total carboxylates), doubling previously achieved values in lactate-fed chain elongation reactors (34–41% nC6) [8, 9] and being comparable to values obtained from ethanol and acetate chain elongation (80–94% MCC per total carboxylates) [38]. It is worth noting that improvements in MCC extraction rates could further reduce potential inhibition by undissociated MCC and enhance chain elongation performance.

After 12 days of extractive fermentation at pH 5.0 (period II-b), however, carboxylate production was reduced by around half although lactate consumption was stable. The missing electrons between substrate conversion and carboxylates production (Fig. 2) may indicate a low recovery of carboxylates by back-extraction during period II-b and III. Alternatively, agitation by stirring in the reactor may have favored mixing of potentially toxic compounds such as acids or triglycerides vesicles that may cause cell membrane disruption [39]. Thus, their migration to the aqueous phase could have partially inhibited bacteria and increased energy demand for solvent tolerance adaptation and cell maintenance [40]. Though some rumen bacteria including chain-elongating bacteria *Megasphaera* *elsdenii* and *Eubacterium* *pyruvativorans* showed improved resistance to linoleic acid in the presence of lactate [41]. Thus, the use of lactate as electron donor may promote extra resilience of *Caproiciproducens*-dominated microbiomes to sunflower oil released components. Measuring LCC concentrations in the aqueous phase of continuous or spiked batch reactors and their effect on fermentation could be done to test these hypotheses. No known LCC degraders were observed in this study [42]. *Sporoanaerobacter*-related species detected at pH 7.0 (period III) have been observed in methane-inhibited reactors degrading monounsaturated LCC with no described role in such microbiomes [43].

Comparative stoichiometric analysis of non-extractive (periods I-a to I-c) and extractive (period II-a) chain elongation in R1 indicates that n-caproate selectivity was improved due to decreased propionate and n-butyrate yields (Additional file 1: Table S5). Extractive chain elongation resulted in almost halved net acetate consumption compared to non-extractive conditions with molar ratios of lactate-to-acetate consumed being 6.4 and 11 during non-extractive and extractive chain elongation in R1, respectively, showing the metabolic flexibility of lactate-elongating microbiomes. n-Caproate yields obtained in this study were 0.29–0.32 mol nC6 mol lactate_consumed_^−1^, similar to yields reported for pure cultures converting lactate and acetate (0.26 mol nC6 mol lactate_consumed_^−1^) [44].

### MCC extraction with sunflower oil

Sunflower oil showed to be biocompatible with chain-elongating microbiomes as similar amounts of carboxylates were produced compared to non-extractive and extractive fermentation with oleyl alcohol (“Sunflower oil and oleyl alcohol biocompatibility and carboxylates extraction efficiency in batch extractive chain elongation”). Higher n-caproate production compared to oleyl alcohol was probably related with the high sunflower oil specificity for n-caproate extraction even when n-butyrate was the main fermentation product in batch extractive fermentation. Sunflower oil reached n-caproate recovery of 45% with only 6% n-butyrate extracted and the n-caproate specificity attained here (55%) is similar to ionic liquids using synthetic chain elongation effluent (53%) [7]. To improve MCC recovery, sunflower oil could be modified with reactive extractants or mixed with more efficient solvents. A mixture of sunflower oil and 1-octanol showed n-caproate recoveries of ~ 95 and ~ 50% at pH 4.5 and 5.5, respectively, from equimolar C2–nC6 solutions [45]. However, other short-chain carboxylates (C3–nC5) were extracted together with n-caproate. Thus, the modifiers should not reduce extraction selectivity substantially and must be compatible with the final application as well. Oleyl alcohol allowed higher n-caproate recovery with selectivities at pH 6.0–6.5 similar to sunflower oil but declining at lower pH conditions (pH ~ 5.0) (lactate vs food waste experiments).

Minimal extraction of substrates and intermediates is important to sustain efficient extractive chain elongation processes. In continuous biotic and abiotic experiments at pH 5.0, sunflower oil exclusively extracted MCC from the fermentation broth. The fact that lactate was not extracted in our experiments is in line with previous reports, where non-reactive extraction of lactic acid showed very low efficiency [15]. For ethanol-based chain elongation, however, the use of vegetable oils could result in ethanol extraction [18] and reduced bioprocess efficiency. The concentration of n-caproate and n-caprylate in MCC-enriched sunflower oil was increased by a factor of ~ 11 (72 g L^−1^) and ~ 38 (3 g L^−1^), respectively, with respect to aqueous concentrations in non-extractive chain elongation (6.6 g nC6 L^−1^, 0.08 g nC8 L^−1^) (R1). This concentrations equal to MCC contents of 8.3 wt% MCC, slightly higher than n-caproate loads reported for anion-exchange resins (≤ 6.2 wt%) [11].

Recovery of MCC was ~ 18% for n-caproate and 100% for n-caprylate during continuous extractive chain elongation at pH 5.0. This product recovery in continuous systems may be significantly enhanced by process operation/design to improve MCC flux to the organic phase. This could be accomplished, for instance, with higher aqueous-organic interface areas (e.g., membrane-supported pertraction) [8, 20] and solvent/broth recirculation [14]. Conducting chain elongation at lower biocompatible pH (e.g., pH 4.5) would increase the fraction of hydrophobic undissociated carboxylic acids and their extraction flux. However, this may reduce microbial activity and chain elongation rates [34]. MCC extraction specificity may also be affected at lower pH, since conditions of pH ≤ p*K*_a_ have been observed to promote SCC extraction rates to similar values as for MCC in membrane contactors [20]. MCC recovery could also be improved by refreshing the sunflower oil sooner, resulting in lower MCC content but higher extraction flux.

### Potential of MCC-enriched vegetable oils as feed additives

Supplementation of vegetable oils to livestock diet has been intensively researched as a device for influencing their physiology and gut microbiomes. Oil addition can result in faster growth, improved feed efficiency and in modification of the derived food products [25, 26]. On the other hand, MCC are metabolized differently than LCC [46] with beneficial effects in animal health and growth performance [27, 47]. Environmental benefits might also be achieved using LCC and MCC due to their effectivity at reducing methane emissions in ruminant animals [28, 29]. Diet supplementation with 2–5 wt% of LCC (with respect to dry matter [DM] in the feed) might result in improved feed efficiency and growth in ruminants and swine [25, 26]. Similar positive effects such as weight gain, feed efficiency and gut health are observed with MCC addition to swine diet in the range of 0.2–1 wt% DM [27]. In addition, adding MCC at 0.12–0.15 wt% DM shows therapeutic effects under stressful conditions (e.g., pathogen infections, weaning) by improving gut health, immune responses and survival rates in swine [27]. MCC can also be used against fish pathogens [48]. Since the MCC doses required to show significant effects are one order of magnitude lower than those of LCC, a product with 5–10 wt% MCC in vegetable oil could be suitable as feed additive. These values are comparable to the MCC-enriched sunflower oil produced in this study (6.3–8.3 wt% MCC; n-caproate + n-caprylate), which could potentially be used as biobased functionalized feed additive to improve livestock growth and well-being.

Another distinctive property of MCC is their capability to inhibit pathogens in livestock feed [49] when added at 1–2 wt% DM [27]. Similar MCC dietary proportions seem to be required to achieve methane mitigation (≤ 3 wt% DM), although lauric acid (C12:0) was the only effective MCC [30]. LCC doses for methane mitigation are in the same order (2.6–3.6 wt% DM) [28] which is in line with literature reports showing similar methane mitigation capabilities for lauric acid (MCC), myristic acid (LCC) and linoleic acid (main LCC in sunflower oil) [29]. Since similar MCC/LCC proportions in feed are required for feed pathogen inhibition and methane mitigation, the MCC-rich oil may need to be further enriched with MCC for these applications. However, synergistic antimicrobial activity is observed for different MCC or MCC–LCC mixtures. Antimicrobial activity is higher for MCC blends compared to individual MCC [49] and for MCC mixed with vegetable essential oils [50] or LCC [51]. Using MCC-enriched oils would also avoid the negative impact of unpleasant MCC smell on feed intake [52] and allow higher MCC dietary proportions. The properties of these enriched oils could be modulated by changing the MCC content and individual MCC ratios (n-caproate/n-caprylate). Although the MCC–LCC dietary requirements could be lowered using MCC-enriched vegetable oils, their effectivity for the aforementioned applications requires further research.

### Biotechnological outlook

Using microbially produced carboxylates from low-value organics is getting increasing attention as a way to valorize the growing amounts of residues [53]. Lactate-based chain elongation is promising for upcycling complex organic residues to MCC without adding external electron donors [4, 5]. Here we show that chain elongation from lactate can yield high MCC selectivities. When carboxylates are used as feed additives, livestock-related concerns such as increasing antibiotic resistance [26, 27] and greenhouse gases emissions [30] can be addressed as well. MCC (in their salt form) produced via chain elongation from organic residual materials are now commercially available for animal nutrition [54]. In addition, vegetable oils are also reported to positively influence livestock [25, 26] and they can be used as application-compatible solvents for in situ MCC extraction in chain elongation processes. The use of solvents that are compatible with a specific application may reduce additional equipment, purification steps and salt waste generation. Some of these benefits have been proposed to be achieved in extractive fermentation with engineered strains, where product-enriched solvents are directly used in chemical hydrogenation for aviation fuel applications [22] and reduce process production costs and environmental impact [23]. The potential benefits of application-compatible solvents for the process proposed here should be evaluated considering sustainability aspects related to vegetable oil production [55] and chain elongation processes [56]. Food waste-derived oil could be used as an alternative endogenous solvent that seems to extract MCC [4]. Hazardous compounds possibly present in waste-derived oils [57] may be removed for feed applications. A potential interesting option is to use microbial oils from algae and/or yeast cultures which feature high unsaturated LCC contents and can be obtained using agro-industrial waste streams [58]. Especial attention to extraction of unwanted compounds (e.g., hydrophobic toxins and pollutants) to the organic solvents must be given, since this may hinder the direct use of MCC-enriched solvents. These hydrophobic unwanted compounds might come from the organic residues themselves [57] or be produced by pathogens potentially enriched in the microbiomes. Thus, feedstock selection could be done accordingly to the final application. It is worth mentioning that vegetable oil components can inhibit many known pathogens [39].

A high unsaturated LCC content can promote immune responses in cattle and accumulation of conjugated linoleic acids (CLA) in animal-derived food products [25]. CLA are isomers of linoleic acid considered to have positive physiological effects on human health and are mostly sourced from dairy and meat products [59]. Thus, finding alternative ways to produce CLA could increase their dietary accessibility for human consumption. Microbial CLA production has been reported for different probiotic bacteria [60] and *M.* *elsdenii* [61], a chain-elongating microorganism. Although CLA were not detected in MCC-enriched sunflower oil, vegetable oils enriched with both CLA and MCC could be produced via chain elongation processes for feed or food purposes following pertinent regulations. MCC display differential effects on human health compared to unsaturated LCC [24] and CLA-rich food products are under development [62]. One related alternative of solvent choice is using oleyl alcohol, which is approved as indirect food additive by the FDA and regulates LCC uptake in mammals [63]. Biobased alkyl alcohols (e.g., oleyl alcohol) can be obtained from hydrogenation of plant-derived or microbially produced LCC.

MCC-enriched oils may also find application as biofuels or biochemicals precursors. For instance, MCC-enriched waste-derived oil may be converted into less polluting biodiesels due to their potential lower unsaturation levels compared to conventional biodiesel [64]. LCC triglycerides in the enriched oil may be hydrolyzed to convert the resulting LCC and MCC salts into chemicals or aviation fuels through (non-)Kolbe electrolysis [65]. Sustainable aviation fuels (SAF) produced from waste oils and fats show high CO_2_ savings (> 85%) compared to conventional aviation fuels [66] and ~ 7% of annual aviation fuel consumption could be replaced by SAF obtained from microbially produced MCC [6] in integrated (electro)biorefineries.

## Conclusions

A reactor microbiome was developed for valorisation of residual materials into potential functionalized feed additives. Sunflower oil displaying a high MCC extraction specificity was a biocompatible solvent in extractive lactate-based chain elongation similarly to oleyl alcohol. The use of oleyl alcohol could allow higher carboxylates recovery although with lower MCC extraction specificity. Lactate and intermediate carboxylates (acetate, propionate, n-butyrate) remained in the aqueous phase during continuous extractive chain elongation, while transfer of n-caproate and n-caprylate to sunflower oil relieved apparent undissociated MCC toxicity. Extractive chain elongation microbiomes showed improved substrate conversion, n-caproate production rates and MCC specificities. Uncultured species belonging to the genus *Caproiciproducens* dominated the MCC-producing microbiomes in coordination with hub *Clostridium* and *Lactobacillus* species. The genera *Anaerotignum* and *Lachnospiraceae* UCG-010 were dominant when n-butyrate and propionate were produced at pH 7.0. This shows the relevance of selection pressures (e.g., extractive fermentation, pH) and occurrence of hub organisms to develop effective chain elongation reactor microbiomes.

MCC-enriched solvents produced through extractive chain elongation are potential novel products obtained with simplified downstream processing. The MCC-enriched sunflower oil produced here contained up to 72 g L^−1^ and 3 g L^−1^ n-caproate and n-caprylate, respectively, corresponding to 8.3 wt% MCC. This concentration is high enough for several applications. MCC and LCC contained in the product could act synergistically aiding purposes such as feed pathogens inactivation or livestock gut microbiome regulation. In addition, MCC-enriched solvents could be used in food and biofuels applications.

## Materials and methods

### Extractive batch fermentation with sunflower oil and oleyl alcohol

Carboxylates extraction with sunflower oil was compared against oleyl alcohol in extractive batch experiments. Experiments were carried out in 125 mL serum bottles containing either lactate (3 g L^−1^; ≥ 90% l-lactic acid, VWR) or food waste (10% v/v; 3.46 e^−^ eq L^−1^) with composition described elsewhere [4]. Mineral medium, yeast extract and vitamins were added as described elsewhere [67]. Trace elements were prepared after Zhu et al. [68]. BisTris buffer (~ pH 9.8) was added at 100 mM concentration to reduce pH changes due to biological substrate conversion. The same mineral medium and nutrients were used for both substrates. Initial pH was then adjusted using either 1 M KOH or HCl. An initial pH value of 5.0 was used for lactate experiments to select for n-caproate producers [8], while pH was set to 6.0 in bottles fed with food waste to allow acidification to lactate and subsequent chain elongation [4]. The batch bottles were sealed with a rubber septum and aluminium crimp cap. The headspace was exchanged by filling and vacuum cycles (five times) with 100% N_2_. Inoculum was derived from a continuously stirred tank reactor (CSTR) fermenting food waste to MCC [4], centrifuged, resuspended in oxygen-free demineralized water and injected (2 mL) into the bottles. Fermentation medium volume was 40 mL. Finally, sunflower oil (AH Biologisch Zonnebloemolie, Albert Heijn, the Netherlands) and oleyl alcohol (technical grade, 85% purity, Sigma Aldrich) were injected into the respective bottles to form an organic phase layer on top of the fermentation medium with a solvent-to-medium volume ratio of 20%. Sunflower oil had a room temperature density of 0.902 kg L^−1^ with main components measured to be (g per 100 g carboxylic acids): linoleic acid (C18:2 cis9, 12), 53; oleic acid (C18:1 cis9), 35; octadecanoic acid (C18:0), 3; and palmitic acid (C16:0), 6. Both solvents were tested separately in duplicate experiments. For each substrate, singletons without solvent were done to serve as blank experiments. The bottles were incubated in an orbital shaker at 30 °C and 100 rpm. Gas and liquid samples were taken regularly. Carboxylates were quantified in both aqueous and solvent (after back-extraction) samples.

### Continuous lactate-based chain elongation and extraction with sunflower oil

Continuous experiments were carried out using l-lactate (50% sodium-(S)-lactate, Merck) and acetate (acetic acid > 99%, Sigma Aldrich) as substrates for microbial chain elongation. Experiments were done in two independent 2-L CSTR (R1 and R2) each with a diameter of 10.5 cm and working volume of 1.2 L (Applikon, Schiedam, the Netherlands). Polyurethane foam was used as carrier material (two sheets of 9 cm × 9 cm × 1 cm) positioned at baffles level to alleviate possible limitations in conversion rates related to biomass concentration (Additional file 1: Fig. S8). The reactors were adapted for feed and acid dosing below the overlaid organic phase and for solvent and fermentation broth sampling (Additional file 1: Fig. S8). The medium composition (minerals, trace elements, yeast extract and vitamins) was the same as described in “Extractive batch fermentation with sunflower oil and oleyl alcohol”. l-Lactate was added as electron donor at 40 g L^−1^, acetate at 5 g L^−1^ as electron acceptor and pH was adjusted to 5.0 with 4 M KOH. Fermentation broth was bubbled with N_2_ to ensure anaerobic conditions. The inoculum (30 mL) derived from a chain elongation reactor [4] was the same as for extractive batch experiments. Operational conditions for each period are shown in Additional file 1: Table S6. Temperature (30 °C), HRT (2 days) and stirring speed (80 rpm) were kept constant throughout the experiments. pH was controlled by automatic addition of 1 M HCl. Non-extractive chain elongation was evaluated at pH 5.0 (without solvent addition). Then, sunflower oil was added to evaluate extractive chain elongation keeping the same pH and HRT conditions. Sunflower oil was added through the solvent sampling port on day 99 in R1 and day 100 in R2 to an oil-to-medium volume ratio of 20% v/v. Sunflower oil was laid as a static layer over the fermentation broth, while the fermentation medium was continuously stirred and replenished. After ~ 15 days, the oil was collected out of the reactors manually with the help of a syringe. After adjusting pH in the fermentation broth to 7.0, sunflower oil was added for the second occasion on day 116 to R1 and day 117 to R2. Gas, aqueous and oil phases were sampled every other day. Long-chain carboxylates in sunflower oil (saturated and unsaturated LCC) were monitored during extractive fermentation.

### Abiotic sunflower oil saturation in continuous experiments

The same CSTR setup was used to test extraction of carboxylates into sunflower oil in continuous abiotic experiments. For this, R1 and R2, hereafter referred to as R3 and R4, respectively, were used after the biological continuous experiments (“Continuous lactate-based chain elongation and extraction with sunflower oil”). The reactors were filled with acidified water (pH 2, with HCl) for 3 days with stirring at 150 rpm. This procedure was done twice to clean the reactors. Synthetic effluent was prepared resembling carboxylates concentrations from non-extractive CSTR fermentation effluents. The synthetic effluent was fed to keep an HRT of 2 days, temperature and pH in the reactors were, respectively, controlled at 30 °C and 5.0 (with 1 M HCl). Stirring speed was set at 80 rpm as in the biotic experiments. The synthetic effluent contained (g L^−1^): lactate, 11; acetate, 2.6; n-butyrate, 1.3 and n-caproate, 6.1. Carboxylates (except l-lactate which was added as sodium lactate, (“Continuous lactate-based chain elongation and extraction with sunflower oil”) were added in their acid form. Mineral medium and trace elements were added as in the fermentation experiments. Nitrogen sources were removed from the medium to avoid microbial growth. Therefore, phosphate in NH_4_H_2_PO_4_ was replaced with K_2_HPO_4_ and yeast extract was left out. Synthetic effluent pH was adjusted to 5.0 with 4 M KOH. Carboxylates concentration in aqueous and oil samples was measured every other day.

### Analytical methods

Aqueous and solvent samples were centrifuged at 10,000 rpm (radius, 8.6 cm) for 10 min and stored at − 4 °C before analyses. Solvent samples were mixed with an alkaline solution (0.5 M sodium borate, pH ~ 9.4) to back-extract any carboxylates contained in the solvent. 500 µL of oil sample were placed into a 10 mL serum bottle containing 2 mL of sodium borate solution (1:4 solvent-to-alkali). The mixture was shaken vigorously for 1 min and left 30 min to phase separate. The aqueous phase was then collected for carboxylates quantification. Gas chromatography (GC) was used for headspace gas composition as well as for carboxylates (C2–C8) and alcohols (C1–C6) quantification. Lactate, succinate and formate were measured by means of HPLC [4]. Methods description can be found in Additional file 1.

Total carboxylic acids composition of sunflower oil, including SCC (nC4 and nC5), MCC (nC6–nC12, C10:1 and C12:1) and LCC (saturated, unsaturated and isomeric C13–C24), was analyzed according to the ISO 15885 standard [31]. The analyses were done externally at the Dutch Milk Controlling Laboratory (Qlip B.V., Zutphen, the Netherlands) using gas chromatography (Trace GC Ultra, Thermo Fischer) with FID detection. Results are expressed as grams per 100 g of total carboxylic acids. Raw chemical experimental data are available in the 4TU.ResearchData repository (https://doi.org/10.4121/17086037).

### Microbiome composition

Samples were centrifuged at 15,000 rpm (radius, 8.6 cm) for 10 min and stored at − 20 °C for DNA extraction and sequencing. DNA was extracted from the pellets (PowerSoil DNA isolation kit, QIAGEN) for amplification of the V3–V4 region of 16S rRNA via Illumina sequencing. The primer set used allowed simultaneous amplification of bacterial and archaean 16S rRNA as described elsewhere [69]. DNA sequences were processed as described previously [4]. In short, the DADA2 pipeline was used and the identified ASVs were automatically submitted to the SILVA database (SILVA 138 SSU Ref NR 99) for taxonomic identification. Forward and reverse reads were trimmed at cycles 240 and 220, respectively, based on the quality profiles obtained. Sequences were deposited in the ENA database (https://www.ebi.ac.uk/ena) under the accession number PRJEB42300. Species assignment is based on exact sequence matching. Selected sequences with non-exact match were submitted to NCBI BLAST query (megablast 16S rRNA bacterial and archaean gene sequences) and the percentage of identity is reported. ASVs with ≥ 0.01% of total counts were used for further analyses. Shannon diversity index boxplots were obtained using the InteractiveDisplay package [70]. Distance-based Redundancy Analysis (dbRDA) was carried out using Bray–Curtis dissimilarity with the capscale function from the vegan package [71] and visualized with ggord and ggplot2 [72, 73]. Differential abundance analysis was done at ASV level using CSS normalization and the metagenomeSeq package [74] as described elsewhere [4]. In brief, a Zero-Inflated Gaussian Distribution Mixture Model was applied and obtained *P* values from moderated *t* tests between accessions were adjusted using the Benjamini–Hochberg correction method. Differences in taxa abundance between accessions with adjusted *P* values < 0.05 were considered significant. Non-random co-occurrence networks were created using SparCC correlations between microbial taxa at ASV level. For each network, SparCC correlations were calculated for 100 random selections of the ASV counts table with 100 internal iterations using the sparcc function from the SpiecEasi package [75]. This procedure was repeated 20 times and the resulting sparCC covariance matrixes were averaged. The average covariance matrixes were used to create co-occurrence networks using the qgraph package [76]. Correlations with *P* values < 0.05 were used to build the co-occurrence analyses and correlations with absolute strength values > 0.6 are shown in the networks. Nodes depict ASVs sized according to their betweenness centrality (BC) scores to identify hub bacterial species. Networks properties such as number of edges (correlations), nodes (ASVs), average path length (APL) and transitivity were obtained using the igraph package [77].

### Calculations

Details on the calculations to assess extraction and fermentation performance are given in Additional file 1. Extraction performance was evaluated with reference to the carboxylates distribution ratio (*K*_D_), partition coefficient (*P*), recovery and specificity in each solvent by the end of the incubations. Parameters such as product concentration, selectivity, production rate and conversion efficiency were used to study chain elongation performance. The number of electrons for each molecule is: lactate (12 e^−^ eq mol^−1^), acetate (8 e^−^ eq mol^−1^), propionate (14 e^−^ eq mol^−1^), n-butyrate (20 e^−^ eq mol^−1^), n-valerate (26 e^−^ eq mol^−1^), n-caproate (32 e^−^ eq mol^−1^), n-heptylate (38 e^−^ eq mol^−1^), n-caprylate (44 e^−^ eq mol^−1^), hydrogen (2 e^−^ eq mol^−1^) and methane (8 e^−^ eq mol^−1^).

## Supplementary Information

**Additional file 1: Figure S1.** Metabolites concentration in the aqueous phase during extractive batch fermentation with: (A, D) sunflower oil and (B, E) oleyl alcohol. (C, F) Non-extractive fermentation singletons. Substrates were lactate (A–C) and food waste (D–F). Error bars depict duplicates absolute deviation from the average. **Figure S2.** Carboxylates concentration in solvents during batch extractive fermentation. Error bars depict duplicates absolute deviation from the average. **Figure S3.** Conversion rates for lactate-based chain elongation as measured over time in: (A) R1 and (B) R2. Batch periods correspond to days 46.8–54 in R1; days 47.8–55 and 57–64 in R2. Triangles show DNA sampling days (blue – suspended biomass samples, red – both suspended biomass and biofilm samples). DNA sample from period II-a was taken only for R1. **Figure S4.** Aqueous phase metabolites concentrations and pH over time in: (A) R1 and (B) R2. **Figure S5.** Selective extraction of n-caproate with sunflower oil from continuously fed synthetic medium. n-Caproate concentrations in sunflower oil as measured from back-extracted samples in: (A) R3 and (C) R4. Bubble size depicts extraction flux into the solvent based on cumulative carboxylates concentrations between two contiguous sampling points. Sankey diagrams show the carbon flux (mmol C d^-1^) for (B) R3 and (D) R4. The synthetic effluent contained lactate, acetate, propionate, n-butyrate and n-caproate fed to an HRT of 2 days and aqueous phase pH was controlled at 5.0. Nitrogen sources were left out to avoid microbial growth. **Figure S6.** Carbon flux (mmol C d^-1^) during non-extractive and extractive lactate-based chain elongation in (A–C) R1 and (D–F) R2. Sankey diagrams were built using data of periods I-a to I-b (non-extractive pH 5), period II-a (extractive pH 5.0) and period III (extractive pH 7.0). The unidentified missing carbon was assumed to be assimilated into biomass. **Figure S7.** Principal Components Analysis (PCA) plot of carboxylic acids composition of initial sunflower oil and MCC-enriched sunflower oil at different pH conditions. MCC-enriched sunflower oil samples were taken from both reactors at different times during extractive fermentation with sampling points same as in Figure 3. Carboxylic acids compositions was measured according to the ISO 15885 standard. Variables were scaled and centered for analysis. Top 15 variables are shown with vectors colored according to their contribution to variance in the PCA plot. Concentration ellipses depict confidence intervals with α = 0.05 for groups with n>3. Principal Components Analysis (PCA) was done in R Studio using the prcomp function and visualized using the factoextra package [3]. **Figure S8.** (A) CSTR schematic and (B) picture. 1 – feed tank; 2 – oil sampling port; 3 – polyurethane foam; 4 – redox sensor; 5 – pH sensor; 6 – liquid level sensor; 7 – controller (Biocontroller ADI 1010, Applikon); 8 – stirring engine; 9 – solvent; 10 – fermentation broth; 11 – gas condenser (4°C); 12 – gas sampling port; 13 – gas meter (µFlow, Bioprocess Control); 14 – liquid sampling port; 15 – water jacket; 16 – effluent tank; 17 – acid tank (A). **Table S1.** Net production of carboxylates in extractive fermentation of lactate and food waste after 20 days. **Table S2.** Overview of chain elongation reactor R1 performance under non-extractive (I-a to I-c) and extractive (II-a to III) conditions. **Table S3.** Overview of chain elongation reactor R2 performance under non-extractive (I-a to I-c) and extractive (II-a to III) conditions. **Table S4.** Selected 16S rRNA gene amplicon sequence variants (ASV). **Table S5.** Stoichiometry of lactate-based chain elongation in R1 at pH 5.0 with(out) extraction with sunflower oil. **Table S6.** Overview of continuous chain elongation operational parameters.

## Data Availability

The data sets generated during the current study are available in the 4TU.ResearchData repository (https://doi.org/10.4121/17086037). 16S rRNA gene raw sequences are deposited in the ENA database (https://www.ebi.ac.uk/ena) under the accession number PRJEB42300.
