# Supplementary material for: Reactor microbiome enriches vegetable oil with n-caproate and n-caprylate for potential functionalized feed additive production via extractive lactate-based chain elongation
Source: Biotechnol Biofuels. 2021 Dec 6;14:232. doi: 10.1186/s13068-021-02084-9 (PMC8647473; doi:10.1186/s13068-021-02084-9)
Supplement: Supplementary file 1 — Additional file 1: Figure S1. Metabolites concentration in the aqueous phase during extractive batch fermentation with: (A, D) sunflower oil and (B, E) oleyl alcohol. (C, F) Non-extractive fermentation singletons. Substrates were lactate (A–C) and food waste (D–F). Error bars depict duplicates absolute deviation from the average. Figure S2. Carboxylates concentration in solvents during batch extractive fermentation. Error bars depict duplicates absolute deviation from the average. Figure S3. Conversion rates for lactate-based chain elongation as measured over time in: (A) R1 and (B) R2. Batch periods correspond to days 46.8–54 in R1; days 47.8–55 and 57–64 in R2. Triangles show DNA sampling days (blue – suspended biomass samples, red – both suspended biomass and biofilm samples). DNA sample from period II-a was taken only for R1. Figure S4. Aqueous phase metabolites concentrations and pH over time in: (A) R1 and (B) R2. Figure S5. Selective extraction of n-caproate with sunflower oil from continuously fed synthetic medium. n-Caproate concentrations in sunflower oil as measured from back-extracted samples in: (A) R3 and (C) R4. Bubble size depicts extraction flux into the solvent based on cumulative carboxylates concentrations between two contiguous sampling points. Sankey diagrams show the carbon flux (mmol C d-1) for (B) R3 and (D) R4. The synthetic effluent contained lactate, acetate, propionate, n-butyrate and n-caproate fed to an HRT of 2 days and aqueous phase pH was controlled at 5.0. Nitrogen sources were left out to avoid microbial growth. Figure S6. Carbon flux (mmol C d-1) during non-extractive and extractive lactate-based chain elongation in (A–C) R1 and (D–F) R2. Sankey diagrams were built using data of periods I-a to I-b (non-extractive pH 5), period II-a (extractive pH 5.0) and period III (extractive pH 7.0). The unidentified missing carbon was assumed to be assimilated into biomass. Figure S7. Principal Components Analysis (PCA) plot of carboxylic aci [file 13068_2021_2084_MOESM1_ESM.docx]

Reactor microbiome enriches vegetable oil with n-caproate and n-caprylate for potential functionalized feed additive production *via* extractive lactate-based chain elongation

Carlos A. Contreras-Dávila, Norwin Zuidema, Cees J. N. Buisman and David P.B.T.B. Strik*

Environmental Technology, Wageningen University & Research, Bornse Weilanden 9, 6708 WG, Wageningen, the Netherlands.

***** Correspondence: david.strik@wur.nl

Figures 8; Tables 6.

*Analytical methods*

Lactate (both D- and L-lactate monomers together), succinate and formate were measured by HPLC [1]. Fatty acids and alcohols, i.e. straight-chain fatty acids (C2-C8), isobutyrate, isovalerate (both 2- and 3-methylbutanoic acids together), isocaproate (4- methyl-pentanoic acid) and straight-chain alcohols (C1-C6) were quantified by gas chromatography (Agilent 7890B) using FID-detection at 240°C and a HP-FFAP column (25m x 0.32mm x 0.50µm). The carrier gas was helium at 1.25 mL∙min^−1^ for the first 3 min and 2 mL∙min^−1^ for the rest of the run. Injection volume was 1 µL (split injection 1:25) and injection valve temperature kept constant at 250°C. Oven temperature ramp was: 60°C for the first 3 min; 21°C∙min^-1^ up to 140°C; 8°C∙min^-1^ up to 150°C and constant for 1.5 min; 120°C∙min^-1^ up to 200°C and constant for 1.25 min; 120°C∙min^-1^ up to 240°C and constant for 3 minutes.

Nitrogen, oxygen, methane and carbon dioxide were measured using GC (Shimadzu GC-2010, Japan) equipped with TCD detector and parallel column setup (gas split 1:1) of Agilent PoraBOND Q (50 m x 0.53 mm x 10 µm) and Molsieve 5A (25 m x 0.53 mm x 50 µm). Carrier gas was helium at 22.5 mL∙min^−1^. The oven temperature was 80 °C and TCD 150 °C. Hydrogen was measured with an HP 5890 gas chromatograph by injecting 100 μl of gas-sample on a Molsieve 5A column (30 m × 0.53 mm × 25 μm) with thermal conductivity detection (TCD). The oven temperature was 40 °C and µ-TCD 150 °C. The carrier gas was argon with a flow rate of 20 mL∙min^−1^.

*Calculations*

Solvents extraction efficiency was evaluated by looking at the distribution ratio (K_D_) of the extracted carboxylates (eq. S1); partition coefficient (eq. S2); the recovery of each individual carboxylate (*i*) compared to its total mass production (mg) (eq. S3); and the solvent specificity towards each carboxylate with respect to all extracted carboxylates (eq. S3). Where HA and A^-^ stand for undissociated and dissociated carboxylic acid, respectively, assuming that only HA species are extracted into the solvents. Concentrations are in mg∙L^-1^.

$Distribution ratio \left( K_{D} \right)= \frac{{[{HA}_{i}]}_{org}}{{[{HA}_{i}+A_{i}^{-}]}_{aq}}$ (eq. S1)

$Partition coefficient \left( P \right)= \frac{{[{HA}_{i}]}_{org}}{{[{HA}_{i}]}_{aq}}$ (eq. S2)

$Recovery= 100 \times\frac{{HA}_{i, org}}{{HA}_{i,org}+ {HA}_{i,aq}+ A_{i, aq}^{-}}$ (eq. S3)

$Extraction specificity= 100 \times\frac{{[{HA}_{i}]}_{org}}{\sum_{i}^{j} {[HA]}_{org}}$ (eq. S4)

Chain elongation performance was evaluated based on conversion rates (eq. S5); selectivity (eq. S6) and conversion efficiency (eq. S7).

Conversion rate (e^-^ meq∙L^-1^∙d^-1^) is based on mass balance:

$${Conversion rate}_{i,t}= {accumulation}_{i,t}+{out}_{i,t}-{in}_{i,t}$$

${Conversion rate}_{i,t}=\frac{n_{e,i}}{V_{r}}[\frac{V_{org}}{\Delta t} \left( C_{i,org,t}-C_{i,org,t-1} \right)+ Q_{out}C_{out,i}- Q_{in}C_{in,i}]$ (eq. S5)

where n_e,_*_i_* refers to the number of electrons in compound *i* (liquid and gas metabolites quantified as described in the analytical methods section); V_r_ the reactor working volume (1.2 L); V_org_ the organic phase volume (0.24 L); ∆t the time between sampling time t and t-1 (d); C*_i_*_.org_ the concentration of compound *i* in the organic phase (mmol∙L^-1^); Q_in_ the influent and Q_out_ the effluent flow rates (L∙d^-1^); C_in,_*_i_* the concentration of compound *i* in the influent and C_out,_*_i_* in the effluent (mmol∙L^-1^). The number of carbon atoms *per* mole of product (n_c,_*_i_*) is used instead of n_e,_*_i_* to express conversion rates in mmol C∙L^-1^∙d^-1^.

Electron and carbon selectivity was calculated with respect to liquid and gas metabolites production rates (eq. S6):

${Selectivity}_{i,t}= 100 \times\frac{{rate}_{i}}{\sum_{i}^{j} {rate}_{liq}+\sum_{i}^{j} {rate}_{gas}}$ (eq. S6)

where rate*_i_* is calculated as in eq. S5 for product *i*; rate_liq_ is the production rate of liquid metabolites (propionate, isobutyrate, n-butyrate, isovalerate, n-valerate, isocaproate, n-caproate, n-heptanoate and n-caprylate) and rate_gas_ is the production rate of gaseous metabolites (hydrogen for electron selectivity; carbon dioxide for carbon selectivity). Carbon dioxide (CO_2_) includes gaseous CO_2_ off gas and aqueous inorganic carbon (IC_aq_) species (H_2_CO_3_, HCO_3_^-^ and CO_3_^2-^). IC_aq_ was calculated after de Leeuw et al. (2020) [2] (eq. S8-S9) where: [H^+^] = 10^-pH^; K_¡_ = 10^-6.35^; K_a2_ = 10^-10.33^; and K_H_ = 29.41 atm/M^2^. Methane was sporadically produced at <5 mmol∙L^-1^∙d^-1^ only during CSTRs recovery from biomass wash out.

Conversion efficiency (mol C_product_∙mol C_consumed_^-1^) was calculated based on lactate and acetate consumed (eq. S7):

${Conversion efficiency}_{i,t}= 100 \times\frac{{rate}_{i}}{{rate}_{lactate}+ {rate}_{acetate}}$ (eq. S7)

where rate*_i_*, rate_lactate_ and rate_acetate_ are calculated for product *i*, lactate and acetate, respectively, in mmol C∙L^-1^∙d^-1^ according to eq. S5.

${IC}_{aq}= \frac{\left[ H_{2}{CO}_{3} \right] \times({{[H}^{+}]}^{2} + {K_{a}}_{1} \times\left[ H^{+} \right] + {K_{a}}_{2} \times{K_{a}}_{1})}{{[H^{+}]}^{2}}$ (eq. S8)

$[H_{2}{CO}_{3}]= \frac{\gamma_{{CO}_{2}} \left( - \right) \times P_{tot} (atm)}{K_{H}}$ (eq. S9)

Figure S1. Metabolites concentration in the aqueous phase during extractive batch fermentation with: (**A**, **D**) sunflower oil and (**B**, **E**) oleyl alcohol. (**C**, **F**) Non-extractive fermentation singletons. Substrates were lactate (**A**-**C**) and food waste (**D**-**F**). Error bars depict duplicates absolute deviation from the average.

Figure S2. Carboxylates concentration in solvents during batch extractive fermentation. Error bars depict duplicates absolute deviation from the average.

Figure S3. Conversion rates for lactate-based chain elongation as measured over time in: (**A**) R1 and (**B**) R2. Batch periods correspond to days 46.8-54 in R1; days 47.8-55 and 57-64 in R2. Triangles show DNA sampling days (blue – suspended biomass samples, red – both suspended biomass and biofilm samples). DNA sample from period II-a was taken only for R1.

Figure S4. Aqueous phase metabolites concentrations and pH over time in: (**A**) R1 and (**B**) R2.

Figure S5. Selective extraction of n-caproate with sunflower oil from continuously fed synthetic medium. n-caproate concentrations in sunflower oil as measured from back-extracted samples in: (**A**) R3 and (**C**) R4. Bubble size extraction flux into the solvent based on cumulative carboxylates concentrations between two contiguous sampling points. Sankey diagrams show the carbon flux (mmol C∙d^-1^) for (**B**) R3 and (**D**) R4. The synthetic effluent contained lactate, acetate, propionate, n-butyrate and n-caproate fed to an HRT of 2 days and aqueous phase pH was controlled at 5.0. Nitrogen sources were left out to avoid microbial growth.


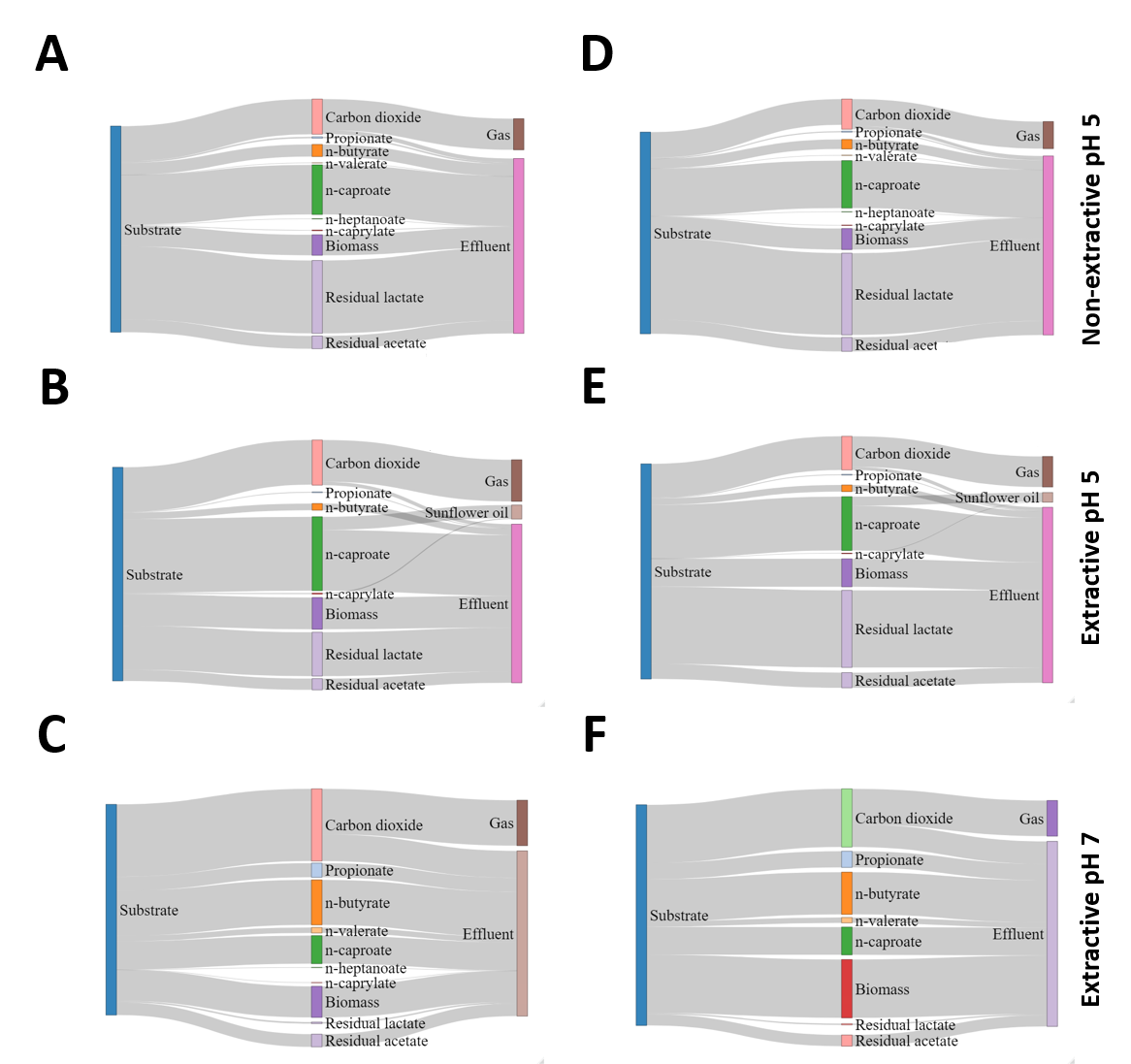


Figure S6. Carbon flux (mmol C∙d^-1^) during non-extractive and extractive lactate-based chain elongation in (**A**-**C**) R1 and (**D**-**F**) R2. Sankey diagrams were built using data of periods I-a to I-b (non-extractive pH 5), period II-a (extractive pH 5.0) and period III (extractive pH 7.0). The unidentified missing carbon was assumed to be assimilated into biomass.3


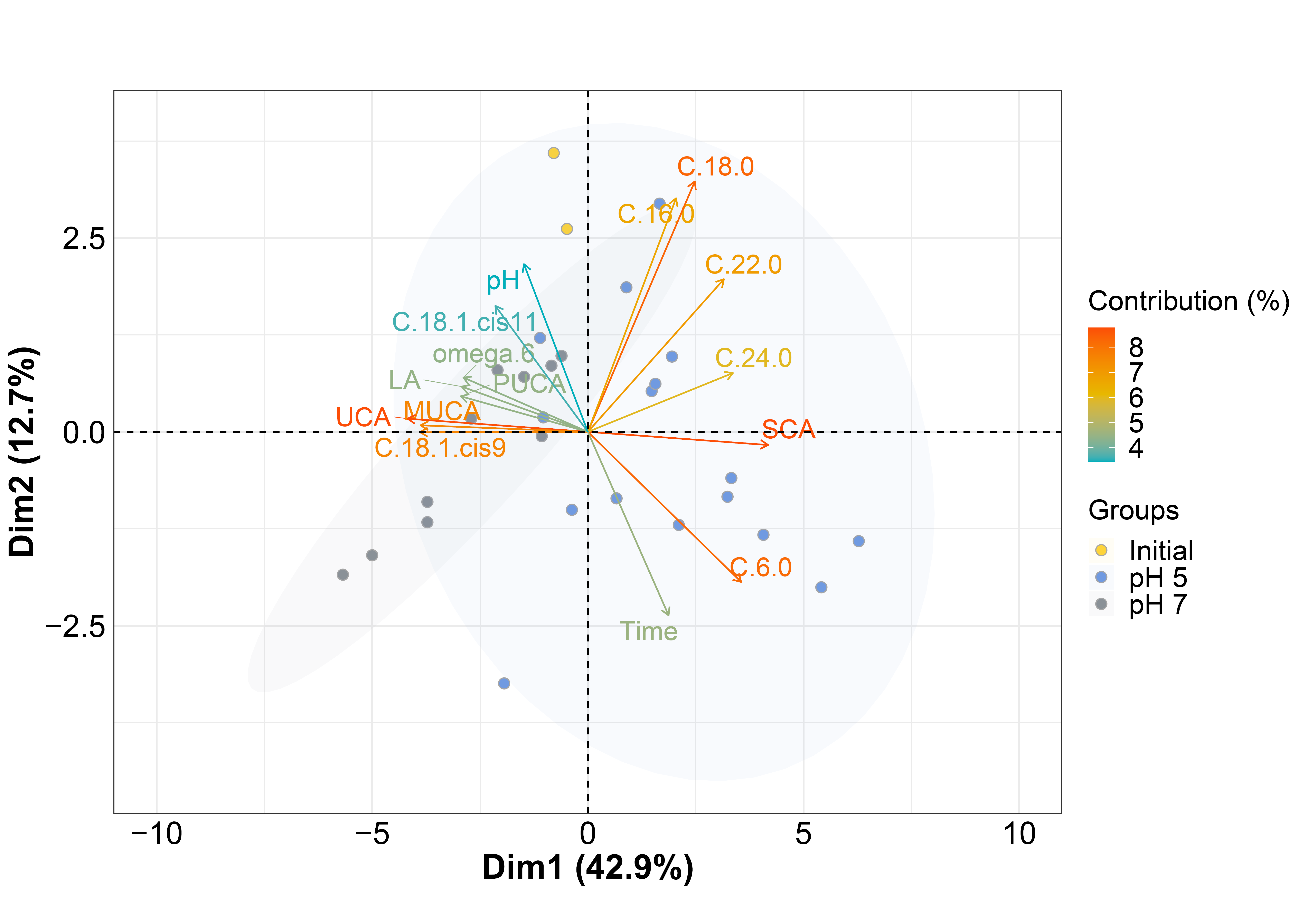


Figure S7. Principal Components Analysis (PCA) plot of carboxylic acids composition of initial sunflower oil and MCC-enriched sunflower oil at different pH conditions. MCC-enriched sunflower oil samples were taken from both reactors at different times during extractive fermentation with sampling points same as in Figure 3. Carboxylic acids compositions was measured according to the ISO 15885 standard. Variables were scaled and centered for analysis. Top 15 variables are shown with vectors colored according to their contribution to variance in the PCA plot. Concentration ellipses depict confidence intervals with α = 0.05 for groups with n>3. Principal Components Analysis (PCA) was done in R Studio using the prcomp function and visualized using the factoextra package [3].


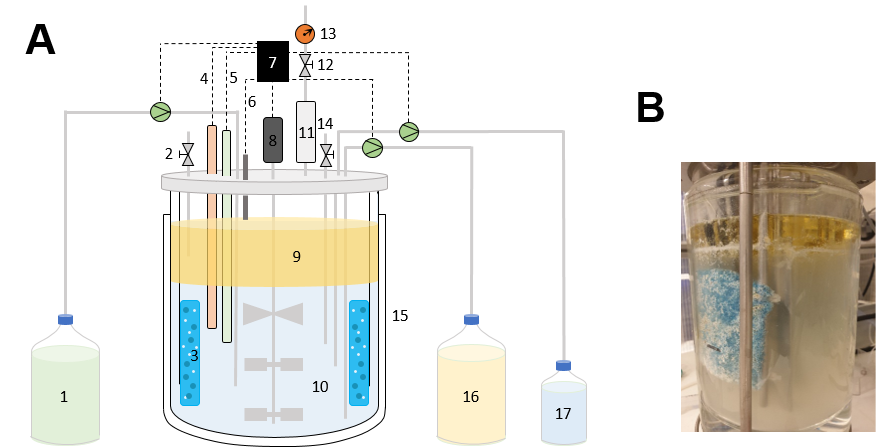


Figure S8. (**A**) CSTR schematic and (**B**) picture. 1 – feed tank; 2 – oil sampling port; 3 – polyurethane foam; 4 – redox sensor; 5 – pH sensor; 6 – liquid level sensor; 7 – controller (Biocontroller ADI 1010, Applikon); 8 – stirring engine; 9 – solvent; 10 – fermentation broth; 11 – gas condenser (4°C); 12 – gas sampling port; 13 – gas meter (µFlow, Bioprocess Control); 14 – liquid sampling port; 15 – water jacket; 16 – effluent tank; 17 – acid tank (**A**).

Table S1. Net production of carboxylates in extractive fermentation of lactate and food waste after 20 days.


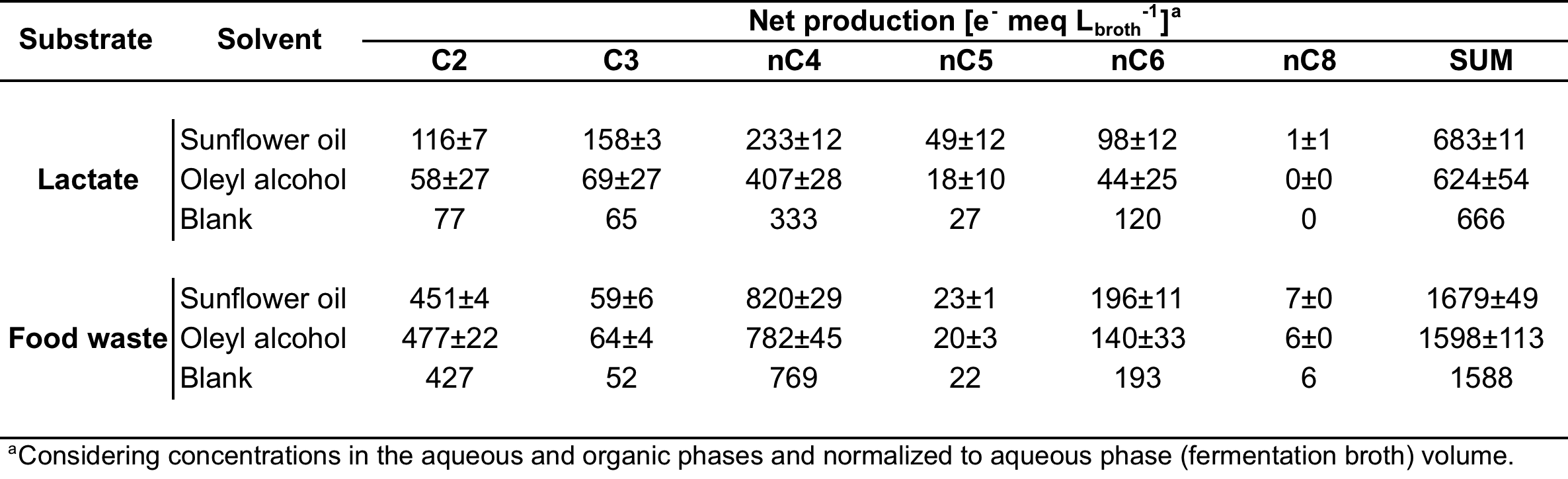


Table S2. Overview of chain elongation reactor R1 performance under non-extractive (I-a to I-c) and extractive (II-a to III) conditions.

Table S3. Overview of chain elongation reactor R2 performance under non-extractive (I-a to I-c) and extractive (II-a to III) conditions.

Table S4. Selected 16S rRNA gene amplicon sequence variants (ASV).

| **ASV** | **Sequence** | **SILVA** |  | **NCBI** | | |
| --- | --- | --- | --- | --- | --- | --- |
|  |  | **Genus** |  | **Strain** | **Coverage [%]** | **Identity [%]** |
|  |  |  |  |  |  |  |
| 1 | GCAGCAACGCCGCGTGAAGGAAGACGGTCTTCGGATTGTAAACTTTTGTACTCAGGGACGATAATGACGGTACCTGAGCAGCAAGCTCCGGCTAACTACGTGCCAGCAGCCGCGGTAATACGTAGGGAGCGAGCGTTGTCCGGATTTACTGGGTGTAAAGGGTGCGTAGGCGGCAGGACAAGTCAGCTGTGAAAACTATGGGCTTAACCCATAGCCTGCAGTTGAAACTGTTCTGCTTGAGTGAAGTAGAGGTAGGTGGAATTCCCGGTGTAGCGGTGAAATGCGTAGAGATCGGGAGGAACACCAGTGGCGAAGGCGGCCTACTGGGCTTTAAC | *Caproiciproducens* |  | [Clostridium] *leptum* strain DSM 753 | 100 | 92.86 |
|  |  |  |  |  |  |  |
| 2 | GCAGCAACGCCGCGTGAAGGAAGACGGTCTTCGGATTGTAAACTTTTGTACCTAGGGACGATAATGACGGTACCTAGGCAGCAAGCTCCGGCTAACTACGTGCCAGCAGCCGCGGTAATACGTAGGGAGCGAGCGTTGTCCGGATTTACTGGGTGTAAAGGGTGCGTAGGCGGCCAAGCAAGTCAGCTGTGAAAACTATGGGCTTAACCCATAGCCTGCAATTGAAACTGTTTGGCTTGAGTGAAGTAGAGGTAGGTGGAATTCCCGGTGTAGCGGTGAAATGCGTAGAGATCGGGAGGAACACCAGTGGCGAAGGCGACCTACTGGGCTTTAAC | *Caproiciproducens* |  | [Clostridium] *leptum* strain DSM 753 | 100 | 92.54 |
|  |  |  |  |  |  |  |
| 4 | GCAGCAACGCCGCGTGAAGGAAGAAGGGTTTCGGCTCGTAAACTTCTATCAACAGGGACGAAAAAAATGACGGTACCTGAATAAGAAGCCCCGGCTAACTACGTGCCAGCAGCCGCGGTAATACGTAGGGGGCAAGCGTTATCCGGAATTACTGGGTGTAAAGGGTGAGTAGGCGGCATGGTAAGTTAGATGTGAAAGCCCGAGGCTTAACCTCGGGATTGCATTTAAAACTATCAAGCTAGAGTACAGGAGAGGAAAGCGGAATTCCTAGTGTAGCGGTGAAATGCGTAGATATTAGGAAGAACACCAGTGGCGAAGGCGGCTTTCTGGACTGAAAC | *Anaerotignum* |  | *Anaerotignum propionicum* strain JCM 1430 | 100 | 98.52 |
|  |  |  |  |  |  |  |
| 7 | GCAGCAACGCCGCGTGAAGGAAGACGGTTTTCGGATTGTAAACTTCTATCAATAGGGACGAAATAAATGACGGTACCTAAATAAGAAGCCCCGGCTAACTACGTGCCAGCAGCCGCGGTAATACGTAGGGGGCAAGCGTTATCCGGAATTACTGGGTGTAAAGGGTGAGTAGGCGGCATGATAAGTAAGATGTGAAAGCCCGCGGCTTAACTGCGGGATTGCATTTTAAACTATTGAGCTAGAGTACAGGAGAGGAAAGCGGAATTCCCAGTGTAGCGGTGAAATGCGTAGATATTGGGAAGAACACCAGTGGCGAAGGCGGCTTTCTGGACTGAAAC | *Lachnospiraceae* UCG-010 |  | *Anaerotignum aminivorans* strain SH021 | 100 | 94.97 |
|  |  |  |  |  |  |  |
| 8 | GCAGCAACGCCGCGTGAAGGAAGACGGTTTTCGGATTGTAAACTTCTATCAATAGGGAAGAAAGAAATGACGGTACCTAAATAAGAAGCCCCGGCTAACTACGTGCCAGCAGCCGCGGTAATACGTAGGGGGCAAGCGTTATCCGGAATTACTGGGTGTAAAGGGTGAGTAGGCGGCATGACAAGTAAGATGTGAAAGCCCGCGGCTTAACTGCGGGATTGCATTTTAAACTGTTGAGCTAGAGTACAGGAGAGGAAAGCGGAATTCCCAGTGTAGCGGTGAAATGCGTAGATATTGGGAAGAACACCAGTGGCGAAGGCGGCTTTCTGGACTGAAAC | *Lachnospiraceae* UCG-010 |  | *Anaerotignum aminivorans* strain SH021 | 100 | 94.97 |
|  |  |  |  |  |  |  |
| 9 | GCAGCAACGCCGCGTGAGTGAAGAAGGTTTTCGGATTGTAAAGCTCTGTCATCTGGGACGATAATGACGGTACCAGATGAGGAAGCCACGGCTAACTACGTGCCAGCAGCCGCGGTAATACGTAGGTGGCAAGCGTTGTCCGGAATTACTGGGCGTAAAGGGTGCGCAGGCGGACATTTAAGTGAGATGTGAAAGACCCGGGCTTAACTTGGGCAGTGCATTTCAAACTGGATGTCTGGAGTGCAGGAGAGGAGAACGGAATTCCTAGTGTAGCGGTGAAATGCGTAGAGATTAGGAAGAACACCAGTGGCGAAGGCGGTTCTCTGGACTGTAAC | *Clostridium luticellarii* |  | *Clostridium luticellarii* strain FW431 | 100 | 100 |
|  |  |  |  |  |  |  |
| 10 | GCAGCGACGCCGCGTGAGCGATGAAGGTTTTCGGATCGTAAAGCTCTGTCCTAAGGGACGATAATGACGGTACCTTAGGAGGAAGCCCCGGCTAACTACGTGCCAGCAGCCGCGGTAATACGTAGGGGGCGAGCGTTGTCCGGATTTATTGGGCGTAAAGGGTGCGTAGGCGGCCTTGTAAGTCAGATGTGAAATCTCACGGCTTAACCGTGGTAAGCATTTGAAACTGTGAGGCTTGAGTACAGGAGAGGAGAGTGGAATTCCTAGTGTAGCGGTGAAATGCGTAGATATTAGGAGGAATACCAGTGGCGAAGGCGACTCTCTGGACTGTAAC | Unclassified *Sporanaerobacter* |  | *Sporoanaerobacter acetigenes* strain DSM13106 | 100 | 100 |
|  |  |  |  |  |  |  |
| 11 | GCAGCAACGCCGCGTGAGTGAAGAAGGTTTTCGGATTGTAAAGCTCTGTCATCTGGGACGATAATGACGGTACCAGATGAGGAAGCCACGGCTAACTACGTGCCAGCAGCCGCGGTAATACGTAGGTGGCAAGCGTTGTCCGGAATTACTGGGCGTAAAGGGTGCGCAGGCGGACATTTAAGTGAGATGTGAAATACCCGGGCTTAACCCGGGCAGTGCATTTCAAACTGGGTGTCTGGAGTGCAGGAGAGGAGAACGGAATTCCTAGTGTAGCGGTGAAATGCGTAGAGATTAGGAAGAACACCAGTGGCGAAGGCGGTTCTCTGGACTGTAAC | *Clostridium luticellarii* |  | *Clostridium luticellarii* strain FW431 | 100 | 98.8 |

Table S5. Stoichiometry of lactate-based chain elongation in R1 at pH 5.0 with(out) extraction with sunflower oil.

Table S6. Overview of continuous chain elongation operational parameters.

**REFERENCES**

1. Contreras-Dávila CA, Carrión VJ, Vonk VR, Buisman CNJ, Strik DPBTB. Consecutive lactate formation and chain elongation to reduce exogenous chemicals input in repeated-batch food waste fermentation. Water Res. 2020;169:1–10.

2. de Leeuw KD, de Smit SM, van Oossanen S, Moerland MJ, Buisman CJN, Strik DPBTB. Methanol-Based Chain Elongation with Acetate to n-Butyrate and Isobutyrate at Varying Selectivities Dependent on pH. ACS Sustain Chem Eng. 2020;8:8184–94.

3. Kassambara A, Mundt F. Package “factoextra.” 2020.

3. Zhu, X.; Zhou, Y.; Wang, Y.; Wu, T.; Li, X.; Li, D.; Tao, Y. Production of High-Concentration n-Caproic Acid from Lactate through Fermentation Using a Newly Isolated Ruminococcaceae Bacterium CPB6. Biotechnol. Biofuels. 2017;10(1):1–12.
